# An Integrative Proteomic Approach to Reveal Altered Signaling Modules During Alzheimer’s Disease Progression in PS19 Tauopathy Mice

**DOI:** 10.1016/j.mcpro.2026.101580

**Published:** 2026-05-05

**Authors:** Eunji Cho, Seulah Lee, Hagyeong Lee, Jaehoon Kim, Yang Woo Kwon, Hyang-Sook Hoe, Dayea Kim, Jong Hyuk Yoon

**Affiliations:** 1 Neurodegenerative Diseases Research Group, Korea Brain Research Institute, Daegu, Republic of Korea; 2 AI-based Neurodevelopmental Diseases Digital Therapeutics Group, Korea Brain Research Institute, Daegu, Republic of Korea; 3 New Drug Development Center, Daegu-Gyeongbuk Medical Innovation Foundation (K-MEDI hub), Daegu, Republic of Korea

**Keywords:** Alzheimer’s disease, dementia, integrative proteomic approach, signaling modules

## Abstract

Alzheimer's disease (AD) is a slowly progressive neurodegenerative disease that is characterized by cognitive, functional, and behavioral impairments. These changes occur owing to the progressive accumulation of extracellular amyloid-beta plaques and intracellular neurofibrillary tangles of hyperphosphorylated tau protein. AD is associated with the dysfunction of several essential neurotransmitter systems, such as dopamine, and impaired neurotransmission. Despite the association of neurotransmitter changes within the brain and AD pathology, in-depth profiling studies on neurotransmitters and their related proteomic changes are limited. This study was conducted to profile and integrate the proteomes and neurotransmitters in seven brain regions of PS19 (Tau P301S) mice according to AD progression between 4 and 7 months. Proteomic analysis revealed significantly altered canonical pathways in various brain regions, including metabolic abnormalities. In the neurotransmitter profile, we found significant alterations in the levels of six neurotransmitters—dopamine, serotonin, homovanillic acid, norepinephrine, 3-methoxytyramine, and 3,4-dihydroxyphenylacetic acid—during AD progression. Using an integrative approach between proteome and neurotransmitter profiles, we found that AD progression-dependent dopamine- and serotonin-related signaling modules are closely related to neurotransmitter changes, especially in the hippocampus and cerebellum. This integrative approach could provide new signaling modules to help understand AD progression and thereby enable improved treatment and clinical outcomes.

## Highlights

• In AD, neurotransmitter systems, such as dopamine, and neurotransmission are impaired.
• We profiled and integrated proteomes and neurotransmitters in the brain of PS19 mice.
• Six neurotransmitters are significantly altered during AD progression.

## Graphical Abstract

**Figure:**
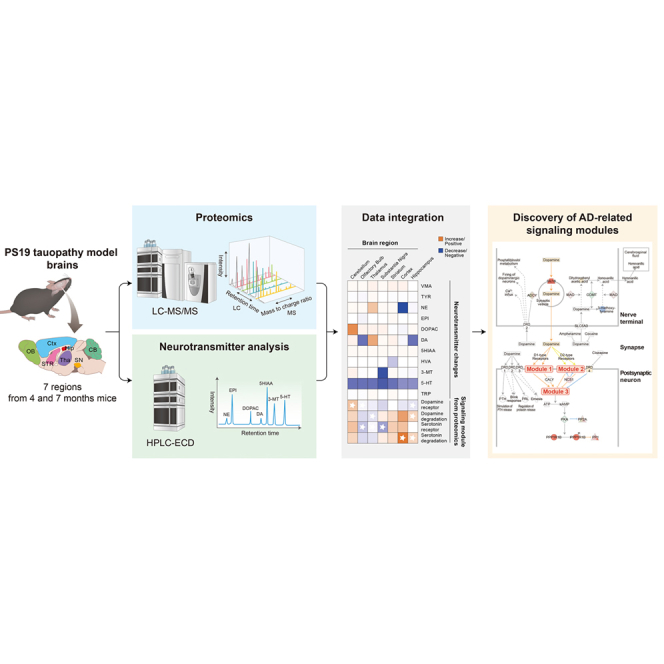

## In Brief

Here, we identified region-specific signaling modules in the PS19 mouse brain that are synchronized with neurotransmitter alterations associated with Alzheimer’s disease. By integratively analyzing proteomic data with disease-relevant molecular features, this work demonstrates the value of multi-omics integration for achieving a more precise and biologically relevant understanding of Alzheimer’s disease.

Alzheimer's disease (AD), a major cause of dementia, is the most common neurodegenerative disorder worldwide and is associated with irreversible cognitive decline (1). More than 55 million people worldwide have AD, and it is estimated that nearly 10 million new cases will be diagnosed every year (http://www.who.int/news-room/fact-sheets/detail/dementia); this estimated incidence is attributed to the progressive increase in the number of patients because of the increasing life expectancy and population aging. AD is pathologically characterized by the accumulation of extracellular amyloid-beta (Aβ) and intracellular neurofibrillary tangles of hyperphosphorylated tau protein (2) and has long been associated with a dysfunctional cholinergic system (3). The cholinergic hypothesis of AD, which highlights acetylcholine as a key neurotransmitter in learning and memory, has been evinced in the research findings of reduced choline acetyltransferase activity and cholinergic neuronal degeneration in the basal forebrain (3, 4). However, acetylcholine deficiency alone cannot fully explain the neuropathological changes in AD (5). Recent studies have revealed that Aβ and tau protein accumulate ≥20 years prior to the onset of AD symptoms (6). Although tau pathology induces cholinergic neuronal degeneration in transgenic mouse models, the exact underlying mechanism remains unclear. Therefore, comprehensive profiling of neurotransmitter dynamics and elucidation of the associated molecular changes are essential for an accurate understanding of the pathomechanisms underlying AD.

Neurotransmitters are chemical substances that mediate neuronal signal transmission and play a crucial role in neurological function and regulation (4). AD is characterized by an imbalance of various neurotransmitters (7, 8). Brain function is mediated primarily through glutamate and gamma-aminobutyric acid (GABA)-based regulation of excitatory and inhibitory signaling (9). Excessive glutamate accumulation can induce neurotoxicity and lead to cognitive decline, particularly memory function (10). As glutamate is possibly synthesized from glucose and is associated with brain energy metabolism, in AD, there is a potential tendency for decreased glutamate levels (11, 12). Compared with healthy controls, individuals with AD generally have reduced GABA levels in the frontal, parietal, and temporal lobes. Reduced GABAergic activity may impair inhibitory control of neuronal excitability and thereby contribute to an imbalance in neural signaling (13, 14). Furthermore, there is a tendency for reduced levels of dopamine (DA) and norepinephrine (NE), which correlates with neuronal loss and cognitive decline (14, 15). However, NE levels may be elevated in some cases, which obscure the precise role of NE in AD pathogenesis (16). In AD, serotonin (5-HT) levels are predominantly decreased and, compared to healthy controls, patients with mild cognitive impairment have up to 25% lower 5-HT transporter levels (17), which is associated with mood disturbances, appetite changes, and depressive symptoms (17, 18). Nonetheless, the causal relationship between altered neurotransmitter levels and AD onset is unclear. Recent studies have emphasized that changes in neurotransmitter systems may manifest during early-stage AD (19). Alterations in neurotransmitter-related pathways in AD may provide valuable molecular insights into disease progression and thereby have potential utility as early diagnostic indicators. Given the paucity of research on the molecular changes that are associated with neurotransmitter synthesis and degradation, this research area warrants further investigation.

Tau is present in the neuronal axonal compartments and mainly regulates microtubular assembly, stabilization, and axonal transport. Misfolded tau can cause synaptic damage (20). Synaptic dysfunction–associated disruption of neurotransmitter homeostasis has been implicated in the pathophysiology of various neurological disorders, including AD, Parkinson’s disease, autism spectrum disorder, and epilepsy (21, 22). Oligomeric tau confers synaptotoxicity that leads to the loss of excitatory synapses and, in postmortem studies of patients with AD, has been identified in the pre- and post-synaptic terminals within the occipitotemporal cortices (23). Therefore, elucidating the molecular changes in neurotransmitter production and tau-associated degradation in AD could contribute to a better understanding of AD pathology. Compared to patients with α-synucleinopathy, those with four-repeat tauopathy had more severe dopaminergic dysfunction in the DA pathway–related brain regions (24). Furthermore, antipsychotics affected the stages of tau accumulation, which increased, depending on DA levels, in a P301L tau mutation mouse model (25). However, the mechanisms by which misfolded tau accumulation modulates neurotransmitter production and degradation, and whether neurotransmitter imbalance leads to tau accumulation, remain unknown. Therefore, elucidating the molecular changes in the production and degradation of various neurotransmitters, in association with tau, could contribute to a better understanding of AD pathology. The P301S (PS19) tauopathy mouse model—one of the most widely studied models—expresses the P301S mutant form and has a median lifespan of approximately 9 months; synaptic dysfunction and microglial activation are detectable as early as 3 months of age, and these precede the appearance of cognitive impairment and neurofibrillary tangle accumulation, which become evident by 6 months. In contrast, astrocyte activation typically emerges later, at approximately 6 months, and is largely localized to the hippocampus (Hip), amygdala, and entorhinal cortex (Ctx) (26, 27, 28). A recent study demonstrated that 5-HT agonism improved tau pathology in PS19 mice and provided evidence that 5-HT_4_ receptor activation alleviated AD (29). However, to the best of our knowledge, few studies have used a PS19 mouse model to comprehensively explore the neurotransmitter profiles in AD.

In accordance with the progression of AD in 4- and 7-month-old PS19 mice, we aimed to analyze and integrate proteomic changes with the neurotransmitter profiles in seven brain regions. The primary objective of this study was to identify significantly altered canonical pathways in the brain regions by using proteomic analysis and profiling of neurotransmitters. Accordingly, we employed an integrative approach to merge the neurotransmitter profiles with proteomic changes.

## Experimental Procedures

### Animal Housing and Genotyping

Tau P301S (PS19) hemizygous (B6;C3-Tg(Prnp-MAPT∗P301S)/PS19Vle/J; MMRRC stock #008169) and WT littermates were produced by mating with B6C3F1/J (stock #100010) females (Jackson Laboratory). Animals were raised under temperature- and light-controlled conditions (20–23 °C under a 12-h light–dark cycle) and provided food and water *ad libitum*. In this study, we used littermates aged 4 and 7 months. For genotyping, 1-mm ear notch samples were placed into e-tubes containing 200 μl DirectPCR lysis agent (Viagen Biotech Inc.) and boiled at 95 °C for 1 h to release genomic DNA; thereafter, 1 μl lysate was used in each PCR reaction that included primers and AccuPower Taq PCR Master Mix (Bioneer). The amplicons were separated by 1.5% agarose gel electrophoresis and the fragments were observed using a FluoroBox imaging system (Cellgentek). The animal experimental protocol used in this study was reviewed and approved by the Institutional Animal Care Committee (Approval Number IACUC-23-00071).

### Experimental Design and Statistical Rationale

Label-free quantitative proteomics was performed across seven brain regions (olfactory bulb (OB), anterior Ctx, striatum (STR), Hip, thalamus (Tha), substantia nigra (SN), and cerebellum (CB)) using samples collected from 4- and 7-month-old female WT and PS19 mice. For liquid chromatography tandem mass-spectrometry (LC-MS/MS) proteomics, each group comprised *n* = 3 biological replicates per brain region (one biological sample per animal per region); each biological sample was acquired by LC-MS/MS once (technical replicates: one per biological replicate, unless otherwise stated). Neurotransmitter profiling by high-performance liquid chromatography with electrochemical detection (HPLC-ECD) was performed using *n* = 4 biological replicates per group per region. To minimize potential run-order and batch effects, all samples were analyzed in a randomized order for both LC–MS/MS-based proteomics and HPLC-based neurotransmitter measurements. The detailed order of sample acquisition is provided in Supplemental Table S2. Statistical analyses were conducted in GraphPad Prism (version 9.0; GraphPad Software Inc.). Two-group comparisons used unpaired two-tailed *t*-tests. For multiple comparisons, we controlled the family-wise error rate using posthoc procedures selected according to the comparison structure. Tukey’s multiple-comparison test was applied when all pairwise group comparisons were performed using one-way analysis of variance. Sidak’s correction was used for post-hoc tests following two-way analysis of variance to adjust the family of simple-effects comparisons while maintaining greater power than the Bonferroni correction. Thus, the Bonferroni correction was applied for predefined comparisons in analyses with unbalanced group sizes across conditions as a conservative approach to control family-wise error rate. All data are presented as mean ± standard error of the mean, and statistical significance was indicated by ∗*p* < 0.05.

### Brain Tissue Preparation

The female mice were anesthetized with carbon dioxide and intracardially perfused with 0.9% normal saline. The skull and meninges were removed and the brain was extracted, washed with ice-cold PBS, and dissected into seven regions. The dissected tissues were placed in e-tubes, immediately snap-frozen in liquid nitrogen, and stored at −80 °C until further use.

### Histological Analysis

#### Brain Section Preparation

The female mice were anesthetized with carbon dioxide, intracardially perfused with 0.9% normal saline, and fixed with a 4% paraformaldehyde solution (Chembio). The brain was removed, placed in the same fixative solution at 4 °C overnight, and transferred to 30% sucrose. Using a CM1850 Cryostat (Leica), cryoprotected brains were serially sectioned at 40 μm in the coronal and sagittal planes, and the sections were stored at 4 °C in Dulbecco’s phosphate-buffered saline solution containing 0.1% sodium azide. For the histological analysis, brain sections were immunostained as described further. Immunostaining was performed using female mice, and each experimental group consisted of *n* = 3 to 4 mice.

#### 3, 3′-DAB Immunohistochemistry

Briefly, to block endogenous peroxidase activity, brain sections were treated with 0.6% H_2_O_2_ in Tris-buffered saline (TBS; pH 7.5), blocked in TBS-TS (0.1% Triton-X and 5% goat serum in TBS) for 30 min, and incubated with primary anti-AT8 antibody (mouse monoclonal; Invitrogen) in TBS-TS at 4 °C. The sections were then exposed to the appropriate biotinylated secondary goat anti-mouse IgG antibody (Vector Laboratories) at room temperature (RT) for 3 h, incubated in ABC solution (ABC Reagent Elite Kit, Vector Laboratories) at RT for 1 h, and developed using a diaminobenzidine (DAB) solution. The DAB-stained tissues were mounted on slides and incubated for 1 min in Mayer’s hematoxylin solution (Abcam) for counterstaining. After rinsing twice with sterile water, the tissues were treated with a bluing solution for 10 s to fix the dye—from the soluble red form to the insoluble blue form. The slides were then rinsed twice and mounted. Images were captured using a panoramic scanning system (3DHistech) and CKX53 microscope (Olympus).

#### Fluorescence Immunohistochemistry

Brain sections were blocked with TBS-TS for 30 min and incubated overnight with primary antibodies for double immunofluorescence staining, including Drd1 and Drd2, AT8 and TH, and AT8 and TPH2, to visualize dopaminergic and serotonergic neurons and their association with tau pathology. The sections were then washed and incubated with anti-rat and anti-rabbit IgG labeled with Alexa Fluor 488 and Alexa Fluor 568 secondary antibodies (Invitrogen), respectively, at RT for 3 h. Images were obtained using a panoramic scanning system (3DHistech) and STELLARIS 8 confocal microscope (Leica). Detailed information on the primary antibodies, including host species, dilution factors, and sources, is summarized in Supplemental Table S1.

### Electrochemical Analysis for Neurotransmitter Profiling

All standard neurotransmitters and neurochemicals, including vanillylmandelic acid (H0131), NE (A0937), epinephrine (E4375), 3,4-dihydroxyphenylacetic acid (DOPAC; 850217), DA (H8502), 5-hydroxyindole-3-acetic acid (5HIAA; H8876), homovanillic acid (HVA; H1252), 3-methoxytyramine hydrochloride (M4251), 5-HT (H9523), L-tryptophan (TRP; 93659), L-tyrosine (93829), and perchloric acid (311421) were purchased from Sigma-Aldrich. N perchloric acid (0.3 N) was added to each slice of brain tissue, and sonication was performed seven times at 12V. The lysates were centrifuged at 18,000*g* for 10 min at 4 °C. Next, 10 μl of the supernatant was injected into a HPLC-ECD system comprising UltiMate 3000 ECD-3000RS (Thermo Fisher Scientific). Chromatographic separation was achieved on a Hypersil BDS C18 column (3 μm, 3 × 150 mm), comprising a Hypersil BDS guard column (28103-013001, Thermo Fisher Scientific) and UniGuard guard cartridge holder (852-00, Thermo Fisher Scientific), with a Dionex mobile Phase (70-3829, Thermo Fisher Scientific), column temperature of 35 °C, and 0.5 ml/min flowrate. The ECD cell potentials were 250 and 400 mV. Each neurotransmitter peak was identified by its retention time compared to that of the standard reference. The concentration was quantified by comparing the peak areas of the samples and the standard chromatograms. Dionex Chromeleon CDS software (Thermo Fisher Scientific) was used for data acquisition and processing. All measurements were performed using female mice, and each experimental group comprised *n* = 4 mice.

### MS Analysis for Proteomics

#### Sample Preparation

After removal from −80 °C storage, brain tissue samples stored in ice were weighed in equal amounts of 5 to 10 mg. For each tube containing 5-mg tissue, 150 μl ProteaseMax at a concentration of 0.2% (w/v) in 40 mM ammonium bicarbonate was added to the sample. Samples were lysed by sonication at 30% amplitude for 3-s on/10-s off on ice for 3 min and then centrifuged at 15,000*g* for 20 min at 4 °C to obtain a supernatant. An additional volume of 40 mM ammonium bicarbonate was then added to the samples to achieve a final ProteaseMax concentration of 0.05%. Following the bicinchoninic acid protein assay (Thermo Fisher Scientific), 100 μg protein was prepared for subsequent analysis. For trypsin digestion, 10 mM DTT was added to the 100-μg sample vial, and the mixture was incubated at 56 °C for 20 min to facilitate reduction. Subsequently, 20 mM iodoacetamide was added to the sample, which was incubated at room temperature in the dark for 20 min to ensure alkylation. Next, 2 μg trypsin (Promega) was added (1:50 w/w enzyme:protein). For digestion, the samples were incubated for 18 h (overnight) at 37 °C and centrifuged at 16,000*g* for 10 s at 4 °C to obtain a supernatant. The reaction was stopped by precipitating the samples by 0.5% TFA for 5 min at 25 °C. The dried peptides were resuspended in a peptide-loading buffer (0.1% FA in water) for MS analysis.

#### LC-MS/MS Analysis

Peptides were analyzed using a Q-Exactive Plus Hybrid Quadrupole–Orbitrap mass spectrometer interfaced with an EASY-Spray source (Thermo Fisher Scientific). Chromatographic separation of the peptides was undertaken using an UltiMate 3000 RSLC nano system (Thermo Fisher Scientific) equipped with an Acclaim PepMap 100 C18 HPLC column (75 mm × 2 cm, 3-mm nanoviper; Thermo Fisher Scientific) and an EASY-Spray PepMap RSLC C18 Column (75 mm × 50 cm, 2 mm; Thermo Fisher Scientific) as the loading and separation columns, respectively. Peptides were injected into an RS autosampler and separated using a linear gradient of acetonitrile/water containing 0.1% formic acid at a 0.3 μl/min flowrate. The eluent was electrosprayed from the separation column, and a 2.0 kV voltage was applied through the liquid junction of the nanospray source. The peptide mixtures were separated using a 10 to 50% acetonitrile gradient for 80 min. The analyses included a full mass scan in the range of 350 to 2000 m/z and data-dependent tandem mass-spectrometry of the 10 most intense ions from the full mass scan. Mass spectrometry was programmed in the data-dependent acquisition mode. The top N most intense precursor ions from each full MS scan were selected for fragmentation using higher-energy collisional dissociation with a normalized collision energy of 27. MS2 spectra were acquired at a resolution of 17,500, with an automatric gain control target of 3 × 10^5^ and a maximum injection time of 100 ms. The MS2 scan range was automatically determined based on the precursor m/z, with a fixed first mass of m/z 50. Mass spectrometer calibration was performed using the proposed calibration solution according to the manufacturer’s instructions. Tandem mass spectral data were processed using the Proteome Discoverer version 2.41 (Thermo Fisher Scientific). Spectral data were searched against the Mouse UniProt database (release version 2023_07). The analysis workflow included four nodes: Spectrum Files (data input), a Spectrum Selector (spectrum and feature retrieval), Sequest HT (sequence database search), and percolator (peptide spectral match or peptide spectral match validation and false-discovery rate analysis). All identified proteins had a false-discovery rate ≤1%, which was calculated at the peptide level. For high-confidence protein identification, proteins that were supported by at least two unique peptides were retained for downstream quantitative and bioinformatic analyses, and proteins that were identified based on a single unique peptide were excluded from the final dataset. The search parameters allowed for tryptic specificity of up to two missed cleavages, with methylthio-modifications of cysteine as a static modification and methionine oxidation as a dynamic modification. The mass search parameters for +1, +2, and +3 ions included mass error tolerances of 20 ppm and 0.6 Da for precursor and fragment ions, respectively. Label-free quantification was performed using the Minora Feature Detector in Proteome Discoverer 2.4.1 to calculate the quantitative changes in proteins among the experimental groups. MS1 precursor ion intensities were used for quantification, followed by total peptide amount normalization. Retention time alignment and feature matching across runs were handled automatically by the Minora algorithm. The mass spectrometry–based proteomics data were deposited in the ProteomeXchange Consortium via the PRIDE partner repository with the dataset identifier PXD066515.

### Bioinformatics Analysis

The Database for Annotation, Visualization, and Integrated Discovery (DAVID) Bioinformatics Resources and ingenuity pathway analysis (IPA) were used for gene ontology (GO)–based functional annotations and in-depth bioinformatics analysis, respectively. We used DAVID (v2023q3) to functionally annotate lists of differentially expressed proteins that were identified in our proteomics experiment. The protein list of UniProt accession numbers was uploaded to DAVID, with the background list set to all genes in the *Mus musculus* genome. We selected the GO biological process annotation category and performed enrichment analysis using the default DAVID settings. Significantly enriched GO terms were identified using Benjamini–Hochberg-adjusted *p*-values <0.05. Analyses were performed separately for each region. In addition, gene set enrichment analysis (GSEA) was performed to identify pathway-level changes using the quantitative proteomics data. Proteins were ranked based on a signed statistic that integrated both the direction and significance of change (sign(log2 fold change) × −log10(*p*-value)). The ranked protein list was analyzed using the GSEAPreranked module with gene sets obtained from the Molecular Signatures Database (MSigDB). The enrichment significance was evaluated using normalized enrichment scores and false discovery rate (FDR q-values). Gene sets with FDR q-values <0.25 were considered significantly enriched. For IPA, the accession numbers of the identified UniProt proteins were coupled with the normalized fold changes between the WT and TG proteomes and uploaded to IPA using the protein expression criteria. A right-tailed Fisher’s exact test was used to calculate a *p*-value to determine the probability that each biological function and/or disease that was assigned to that network was attributable to chance alone and to calculate a *p*-value to determine the probability that the association between the genes in the dataset and the canonical pathway is explained by chance alone. A z-score was calculated to indicate the likelihood of an increase/decrease in that disease or function, or activation/inhibition of that pathway. The following criteria were used for pathway analysis: z-score cutoff ≥2.0 and −log (*p*-value) >1.3. Protein–protein interaction (PPI) networks were generated based on proteins with log_2_(fold change) values between −1.15 and 2.48.

### Sample Preparation for Soluble and Sarkosyl-Insoluble Fractions and Western Blot Analysis

Western blot analysis was conducted using female mice (*n* = 3/group) to quantify the levels of tau proteins and verify that the PS19 mouse model exhibited the pathological characteristics of AD. The anterior cortical tissues were homogenized in a tissue-homogenization buffer containing 10 mM Tris–HCl (pH 7.5), 0.8 M NaCl, 1 mM EDTA, 10% sucrose, and protease inhibitor cocktail (Roche) at a ratio of 500 μl per 100 mg of tissue. Homogenates were centrifuged at 15,000*g* for 20 min at 4 °C, and the resulting supernatant was collected as the soluble fraction. To isolate the Sarkosyl-insoluble fraction, 1% Sarkosyl was added to the soluble fraction and incubated at 37 °C for 1 h with shaking. The mixture was then centrifuged at 20,000*g* for 1 h at 4 °C, and the supernatant was discarded. The resulting pellet was resuspended in 8 M urea and 2% SDS prepared in homogenization buffer, followed by vortexing, brief sonication, and heating at 90 °C, and then centrifuged at 15,000*g* for 20 min at 4 °C. The final supernatant was used as the Sarkosyl-insoluble fraction for immunoblotting. The protein concentration was determined using a bicinchoninic acid protein assay. Protein samples were combined with an SDS sample buffer (Bio-Rad) containing 10% beta-mercaptoethanol and boiled for 5 min. After protein separation by SDS-PAGE, the proteins were transferred onto polyvinylidene difluoride membranes (Millipore) using a Bio-Rad wet-transfer system. The membranes were then blocked by TBS-T containing 5% skimmed milk for 30 min and incubated with primary antibody against total Tau (Abcam) overnight at 4 °C. The membranes were subsequently washed three times with TBS-T and incubated with anti-rabbit IgG horseradish peroxidase–conjugated secondary antibody (GeneTex) for 1 h at room temperature (approximately 25 °C). Finally, membranes were washed with TBS-T and developed using an enhanced chemiluminescence solution.

### RNA Extraction and qRT-PCR

Phenol-chloroform–based extraction was used to isolate total RNA from the OB, Ctx, STR, Hip, Tha, SN, and CB regions of PS19 mouse brains. All experiments were conducted using female mice, and each experimental group comprised *n* = 8 to 11 mice. For lysis, each brain tissue sample was homogenized in RiboEX (GeneAll) and incubated for 10 min at room temperature. The mixture was separated into phases by adding chloroform, followed by centrifugation at 21,000*g* for 15 min at 4 °C. After collecting the aqueous-phase retaining RNAs, the mixture was precipitated using isopropanol and washed with 80% EtOH. After two washes, extracted RNA was dissolved in nuclease-free water. The concentration and purity of dissolved RNA were measured using a NanoDrop 2000/2000c spectrophotometer (Thermo Fisher Scientific).

After RNA isolation, reverse transcription was performed using SuPrimeScript RT Premix (Genetbio) to synthesize complementary DNA. The mRNA expression was quantified by quantitative real-time polymerase chain reaction (qRT-PCR) using SensiFAST SYBR No-ROX Kit (Meridian) and performed by CFX Duet Real-Time PCR System (Bio-Rad) to validate significant molecules that were associated with signaling modules, which were discovered by using neurotransmitter profiling and proteomics analysis. The primers for targeted genes that were used to validate the selected signaling modules are listed in Supplemental Table S3.

## Results

### Sample Preparation for Proteomic and Neurotransmitter Profiling in Seven Brain Regions of PS19 Mice

**Fig. 1:**
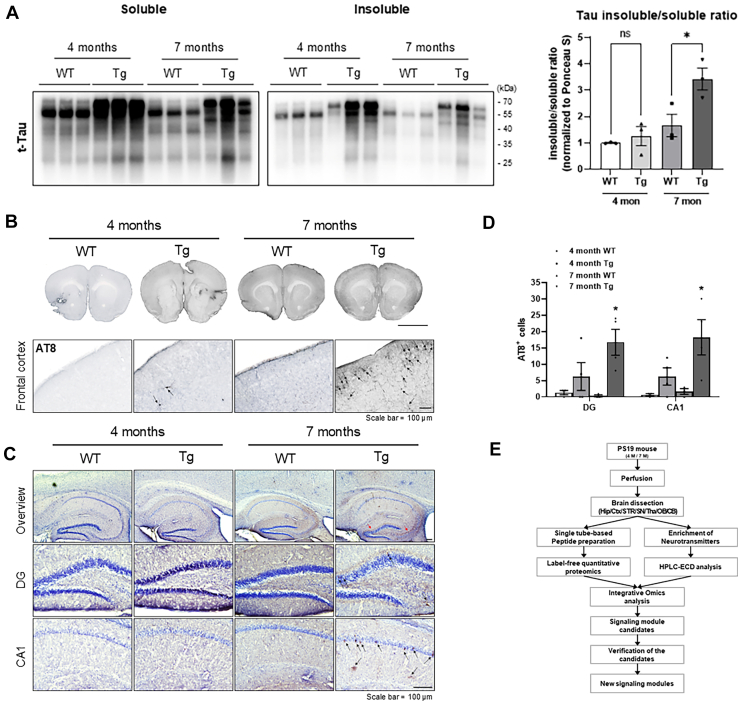
**Pathological characterization of PS19 transgenic (Tg) mice and experimental workflow for integrative proteomics.***A*, representative Western blot images showing the expression levels of total tau proteins in the soluble and Sarkosyl-insoluble fractions extracted from the anterior frontal cortex of WT and PS19 female mice at 4- and 7-month-old. Quantification of band intensities normalized to total protein levels assessed by Ponceau S staining. Data are presented as mean ± SEM; ∗*p* < 0.05 by unpaired two-tailed *t* test. *B*, representative images showing phosphorylated tau (AT8-positive signals) in the frontal cortex (approximately bregma +1.0 to +1.5 mcm) of WT and PS19 female mice at 4- and 7-months of age. Scale bar represents 100 μm. *C*, representative images showing AT8-positive cells in the hippocampus of 4- and 7-month-old female WT and PS19 mice. Scale bar represents 100 μm. *D*, quantification of AT8-positive cells in the hippocampal region. Statistical analysis was performed using one-way ANOVA followed by Bonferroni’s post hoc test. ∗*p* < 0.05; *n* = 3 to 4 per group. Data are presented as mean ± SEM. *E*, workflow for exploring new AD signaling modules using integrative proteomics.

Prior to commencing this study, biochemical analyses were performed to confirm the pathological characteristics of a PS19 AD mouse model. To assess tau aggregation, we biochemically separated soluble and insoluble fractions from cortical samples and measured total tau levels using Western blotting. PS19 mice at 7 months exhibited a significantly increased insoluble-to-soluble tau ratio compared to WT controls, whereas no significant differences were observed at 4 months (Fig. 1*A* and Supplemental Fig. S1). Furthermore, immunohistochemistry revealed the accumulation of phosphorylated tau in PS19 mice. In representative images, no phosphorylated tau was detected in WT mice, whereas a few phosphorylated tau (AT8)-positive signals were observed in the frontal Ctx of 4-month-old PS19 mice. However, in 7-month-old PS19 mice, significantly numerous AT8-positive signals were detected in the frontal Ctx (Fig. 1*B*, black arrow). Moreover, the accumulation of phosphorylated tau was observed in the Hip (Fig. 1*C*). AT8-positive signals were detected at 4 months, with a pronounced increase observed at 7 months. Quantitative analysis further demonstrated a significant elevation in the number of AT8-positive cells in the Hip of 7-month-old PS19 mice compared to WT controls (Fig. 1*D*). Thus, the age-dependent progressive accumulation of phosphorylated tau in PS19 mice demonstrated the successful establishment of an AD mouse model. To explore new AD signaling modules using integrative proteomics, we followed the workflow presented in Figure 1*E*.

### Multi-proteomics of PS19 Brain Regions according to Tauopathy Progression

**Fig. 2:**
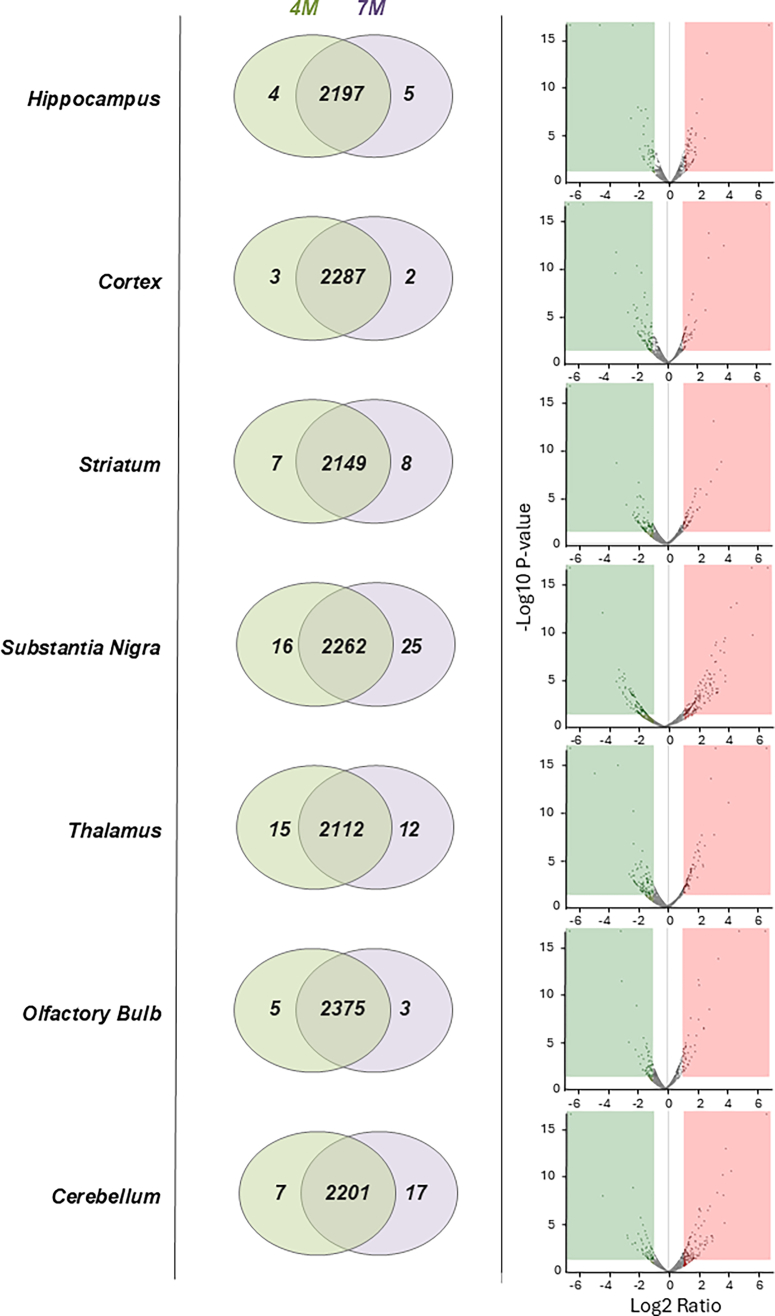
**Proteomic alterations in PS19 brain regions according to tauopathy progression.** Venn diagram and volcano plots showing the identified proteins in PS19 brain regions. The volcano plots display proteins according to log_2_ fold change and –log_10_*p*-value, which highlights differentially expressed proteins that were associated with tauopathy progression.

To evaluate the overall consistency of the proteomic measurements, total ion chromatograms were inspected across all LC–MS/MS runs. The total ion chromatograms showed highly comparable signal intensities and chromatographic patterns among samples from different brain regions and experimental groups, which indicated stable instrument performance and comparable sample loading (Supplemental Fig. S2). Using a quantitative proteomics approach, we profiled the proteomic changes in seven regions of the PS19 mouse brain according to the tauopathy progression. Proteomic analyses identified 2139 to 2383 proteins across the Hip, Ctx, STR, SN, Tha, OB, and CB (Fig. 2*A*). Volcano plots are presented on the side of each Venn diagram to visualize the significantly changed proteins. Detailed protein identification and quantitative information for all identified proteins across the seven brain regions in WT and Tg mice during disease progression (4–7 months) are provided in Supplemental Table S4.

**Fig. 3:**
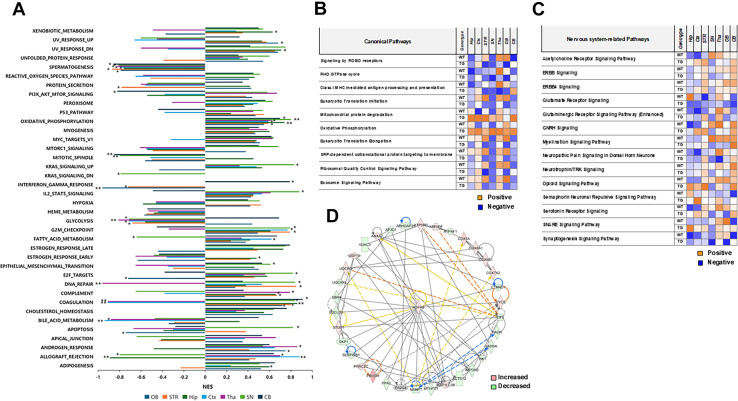
**Functional and network analyses of the regional brain proteomes.***A*, gene set enrichment analysis (GSEA) across brain regions. Normalized enrichment scores (NES) of hallmark gene sets are shown for each brain region. Positive NES values (*right*) indicate upregulated pathways, whereas negative NES values (*left*) indicate downregulated pathways in the comparison. Statistical significance is indicated by asterisks based on false discovery rate (FDR) q-values: ∗FDR q-val <0.25; ∗∗FDR q-val <0.05. *B*, comparative canonical pathway analyses for proteomes using Ingenuity Pathway Analysis (IPA). *C*, comparative nervous system pathway analyses for proteomes using Ingenuity Pathway Analysis (IPA). *Orange* and *blue* indicate canonical pathways with a positive or negative Z-score, respectively, for pathway correlation. Analysis parameters were a Z-score cutoff of 0.5 and a −log(*p*-value) > 1.3. *D*, core target-centered protein–protein interactions (PPIs) in the nervous system. *Red* and *green* indicate that the log_2_ fold increases and decreases under tauopathy progression, respectively. All proteomic datasets were generated from *n* = 3 biologically independent samples per group.

To further characterize pathway-level alterations during tauopathy progression, GSEA was performed using ranked proteomics datasets across seven brain regions (Fig. 3*A*). A total of 34 to 37 gene sets were identified per region. Of these, 16 to 26 gene sets were positively enriched (upregulated pathways), whereas 10 to 20 gene sets were negatively enriched (downregulated pathways), and this demonstrated region-specific differences in pathway enrichment. GSEA revealed distinct pathway alterations across brain regions. Upregulated pathways were predominantly associated with immune-related processes, coagulation, and mitochondrial oxidative phosphorylation, with significant enrichment of coagulation (e.g., Hip, FDR = 0.046; CB, FDR = 0.051) and oxidative phosphorylation pathways (e.g., Hip, FDR = 0; SN, FDR = 0.008) in multiple regions. In contrast, several pathways were significantly downregulated, including those of DNA repair, glycolysis, and mitotic spindle, which indicated impaired genomic maintenance and energy metabolism. For example, DNA repair was significantly reduced in the Tha (FDR = 0.002), whereas glycolysis was decreased in the Ctx (FDR = 0.02). Notably, the concurrent upregulation of oxidative phosphorylation and downregulation of glycolysis suggests a metabolic shift during disease progression. In addition, the dysregulation of cell cycle–related pathways further supports the presence of aberrant cellular responses that are associated with neurodegeneration.

To identify new signaling modules that were involved in tauopathy progression in the PS19 mouse brain, we performed pathway analysis using IPA. In the nonbiased analysis, we compared aging-related changes within each experimental group, WT and PS19 mice, between 4 and 7 months of age. The 10 most significantly altered canonical pathways involved signaling by ROBO receptors, RHO GTPase cycle, class I MHC-mediated antigen processing and presentation, eukaryotic translation initiation, mitochondrial protein degradation, oxidative phosphorylation, eukaryotic translation elongation, SRP-dependent cotranslational protein targeting to membrane, ribosomal quality control signaling pathway, and exosome signaling pathway (Fig. 3*B*). These included various categories of signaling pathways that were related to the nervous system and energy metabolism. Furthermore, the results showed different aging-related correlation patterns (positive/negative) between WT and PS19 littermates (4-month-old vs. 7-month-old). In the nervous system, 14 pathways were significantly affected by aging (Fig. 3*C*). However, these results did not show similar aging-related correlation patterns between WT and PS19. Interestingly, Tg mice that had neurodegenerative aspects did not show a relatively higher negative correlation pattern than the WT mice, as expected. Instead, compared with the WT mice, more positive correlations were observed depending on the brain region. As an extension of the research on the nervous system, including neurological diseases, we analyzed the PPIs of the significant proteins that were involved. This revealed that HSPA8, which was recently been identified as an amyloidase, plays a crucial role in PPIs (Fig. 3*D*) (30). Taken together, these results indicate that aging-related proteomic changes in brain regions have different signaling properties, such as more activated signaling pathways in Tg mice, that are independent of typical observations. Several differences from WT mice were observed regarding the Tg proteome changes during tauopathy progression. This indicates that an integrative study that uses the profiles of neurometabolites is essential to further characterize signaling modules according to the progression of tauopathy in PS19 mice.

### Profiling of Neurotransmitter Changes according to Tauopathy Progression

**Fig. 4:**
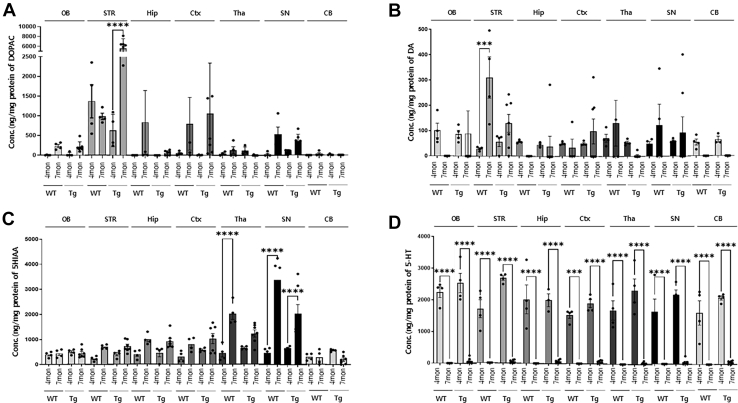
**Quantitative profiling of seven neurotransmitters across brain regions in 4- and 7-month-old in WT and PS19 mice.***A*, DOPAC (3,4-dihydroxyphenylacetic acid), (*B*) DA (dopamine), (*C*) 5-HIAA (5-hydroxyindoleacetic acid), and (*D*) 5-HT (serotonin or 5-hydroxytryptamine) levels were measured in seven brain regions—olfactory bulb (OB), striatum (STR), hippocampus (Hip), cortex (Ctx), thalamus (Tha), substantia nigra (SN), and cerebellum (CB)—WT and PS19 mice at 4-and 7-month-old. Each group consisted of *n* = 3 female mice. Data are presented as mean ± SEM. Statistical significance was determined using two-way ANOVA followed by Sidak's test. ∗∗∗*p* < 0.001, and ∗∗∗∗*p* < 0.0001.

To elucidate the changes in the neurotransmitter profiles that are associated with tauopathy progression, we used HPLC-ECD to measure a total of 11 distinct neurotransmitters and neurochemicals. These neurotransmitters and neurochemicals were analyzed in the same brain regions as those utilized in the proteomic analysis—specifically, the Hip, Ctx, STR, SN, Tha, olfactory, and CB. Initially, standard solutions of neurotransmitters and neurochemicals were simultaneously analyzed within a 10-min runtime to establish a calibration curve. The concentrations of neurotransmitters and neurochemicals were normalized to the protein content. We assessed changes in neurotransmitter and neurochemical levels in both WT and PS19 mice across seven brain regions at 4 and 7 months of age. Notably, differences were observed in the representative neurotransmitters, including DA, DOPAC, 5-HT, and 5HIAA between 4- and 7-month-old mice (Fig. 4). Both the WT and Tg groups are shown in each panel of Figure 4, and these enable a direct comparison of age- and genotype-dependent changes in neurochemical profiles across distinct brain regions. As shown in Figure 4*A*, the differences in DOPAC levels between WT mice at 4 and 7 months of age can be considered aging related. DOPAC was detected in significant amounts in the STR at 4 months, whereas it was present in minimal quantities in other brain regions. At 7 months, DOPAC was detected in all regions; however, there was no statistically significant difference compared with the levels observed at 4 months. In contrast, in PS19 mice (Fig. 4*A*), DOPAC levels significantly increased in the STR between 4 and 7 months of age, which showed a pattern that diverged from that in WT mice. Furthermore, we observed a significant increase in DA levels within the STR of WT mice from 4 to 7 months of age (Fig. 4*B*). However, in PS19 mice, low levels of DA were consistently detected in all the examined regions, whereas 5-HT levels were undetectable across all examined brain regions in the 4-month-old and 7-month-old age cohorts (Fig. 4*D*). Excess intracellular 5-HT is sequentially degraded by monoamine oxidase (MAO) and aldehyde dehydrogenase, which leads to 5HIAA accumulation (31). The results indicated that the 5HIAA concentration increased in all measured regions in WT mice, with a significant increase observed particularly in the Tha and SN (Fig. 4*C*) that suggested a region-specific shift in serotonergic metabolism. In contrast to the pattern of 5HIAA, 5-HT levels in PS19 mice (Fig. 4*D*) exhibited a pervasive decreasing trend across all regions as the tauopathy progressed, which indicated a potential dysregulation of serotonergic signaling in the context of tauopathy. Figure 4 collectively shows genotype- and age-dependent alterations in four representative neurotransmitters and their metabolites (DOPAC, DA, 5HIAA, and 5-HT), which reflect neurochemical changes related to both normal aging and tauopathy progression.

Concentrations of the remaining seven neurotransmitters and neurochemicals are shown in Supplemental Fig. S3. Vanillylmandelic acid levels were comparable between WT and PS19 mice at 4 months of age, with no significant differences observed. Notably, NE was detected exclusively at 4 months in both WT and PS19 mice, whereas epinephrine was uniquely detected at 7 months, which highlights a contrasting pattern. A significant increase in 3 MT was observed in the STR at 7 months, whereas HVA was detected at very low levels solely in the STR. Tyrosine is involved in the production and release of DA through enzymatic reactions, whereas TRP is associated with 5-HT and exhibits the opposite trend. Both TYP and TRP showed a similar sharp increase at 7 months in both WT and PS19 mice. The increase in TYP levels was more pronounced with AD progression than with aging (31).

### Integrative Proteomics Analysis to Discover New Signaling Modules of Tauopathy Progression

**Fig. 5:**
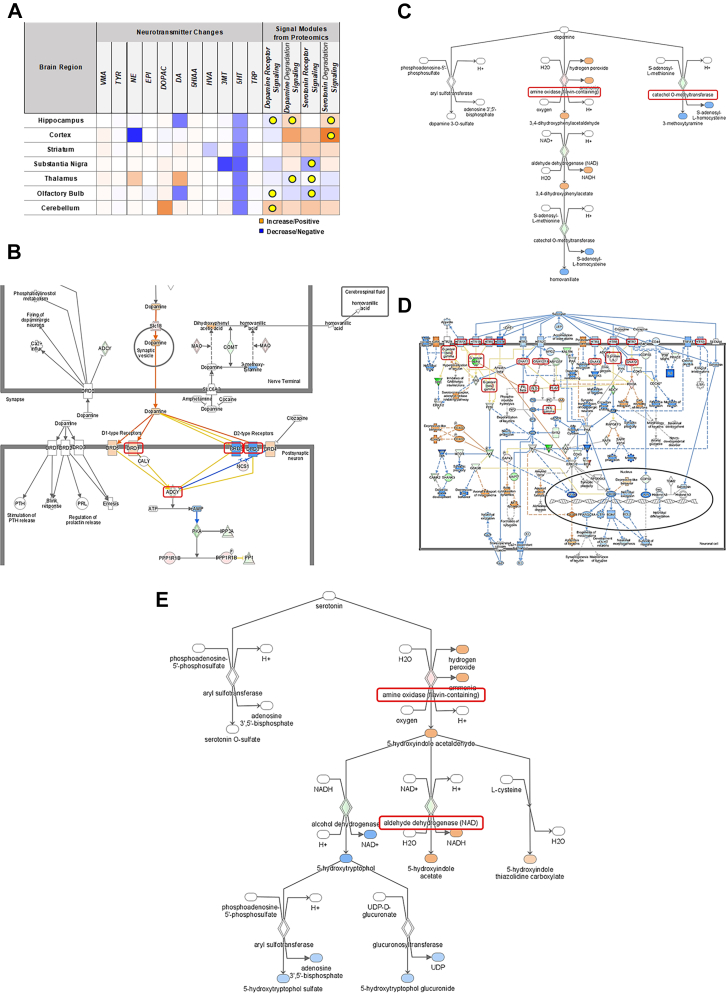
**Integrative proteomics analysis to discover new signaling modules of tauopathy progression.***A*, the panel of the integrative proteomic analysis based on brain region–specific quantitative proteomic data and quantitative profile of neurotransmitters. The analysis compares age-dependent changes (from 4 to 7 months) in both WT and PS19 mice. *Orange* indicates increased level of neurotransmitters in the neurotransmitter changes section and positive Z-score for pathway correlation in the signal modules from proteomics section, respectively. *Blue* indicates decreased level of neurotransmitters in the neurotransmitter changes section and negative Z-score for pathway correlation in the signal modules from proteomics section, respectively. *Yellow* circles represent signaling modules that show consistent directional correlation between neurotransmitter changes and proteomic pathway activity highlighting pathways with strong concordance in both omics layers. *B*, signal transduction pathway of DA receptors signaling. *C*, signal transduction pathway of DA degradation signaling. *D*, signal transduction pathway of 5-HT receptor signaling. *E*, signal transduction pathway of 5-HT degradation signaling. *Red rectangular* indicates the selected key proteins in each pathway.

To discover new signaling modules related to AD progression (4–7 months in PS19 mice), we integrated brain region–specific quantitative proteomic data and quantitative profiles of neurotransmitters and neurochemicals. We first extracted signaling modules related to the signal transduction of neurotransmitters from the pathway analysis results. Moreover, DA-related signaling and 5-HT–related signaling have been discovered. The quantitative profiles of the 11 neurotransmitters and neurochemicals in each brain region were then plotted. Finally, we selected signaling modules based on certain criteria, including correlation with the activation of signaling pathways as well as changes in key regulators of each discovered pathway. Integrative proteomic analysis revealed that the Hip of PS19 mice demonstrated changes in DA- and 5-HT-–related signaling modules accompanying neurotransmitter changes according to tauopathy progression (Fig. 5*A*). The 5-HT–related signaling module (5-HT degradation signaling) in the Ctx of PS19 mice demonstrated changes. The Tha and OB of PS19 mice changed their DA-related signaling modules according to tauopathy progression. The CB of PS19 mice changed both DA- and 5-HT–related signaling modules. To understand the molecular pathways and identify possible molecular targets, signaling transduction pathways, including quantitative proteomic information, are depicted. In DA receptor (Drd) signaling, key proteins such as DA receptors, including Drd1, Drd2, Drd3, Drd4, and Drd5, changed their expression and activation levels according to tauopathy progression (Fig. 5*B*). In DA degradation signaling, key proteins, such as Mao and catechol-O-methyltransferase (Comt), were identified, and their expression levels changed with tauopathy progression (Fig. 5*C*). In the 5-HT pathway, the expression and activation levels of key proteins, including 5-HT receptors, G protein-coupled receptor subunits, and phospholipases, changed in 5-HT receptor signaling (Fig. 5*D*). 5-HT degradation signaling involves changes in aldehyde dehydrogenase (Aldh) levels with MAO, according to the progression of tauopathy (Fig. 5*E*). A list of proteins involved in the selected signaling modules is presented in Supplemental Table S5.

### Validation of the Target Proteins and Genes of the Signal Modules Discovered in This Study

**Fig. 6:**
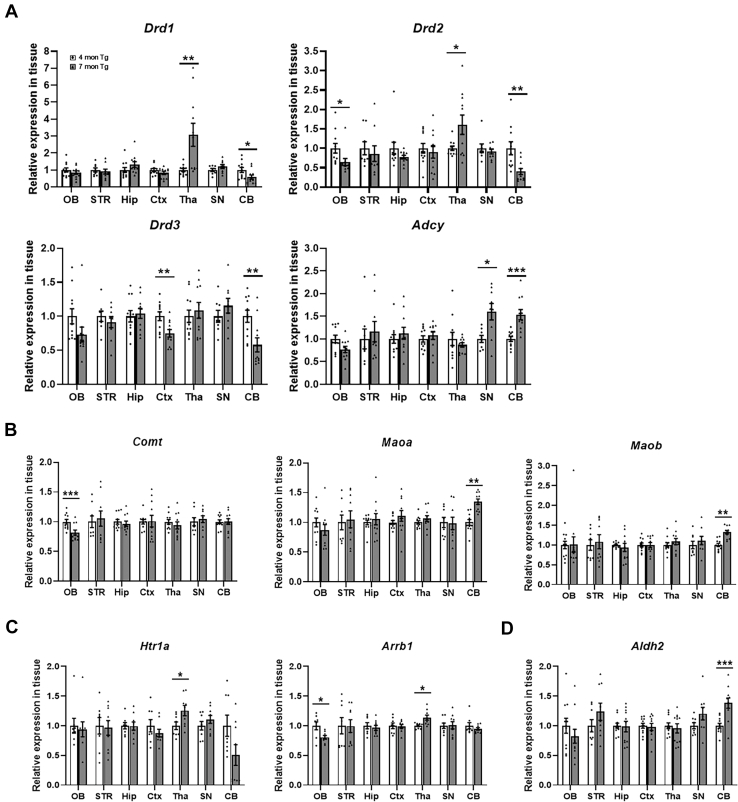
**Validation of target genes of the discovered signaling modules of tauopathy progression.***A*–*D*, relative mRNA expression levels of target molecules involved in the DA receptor signaling pathway (*A*), DA degradation pathway (*B*), 5-HT receptor signaling pathway (*C*), and 5-HT degradation pathway (*D*) in relation to tau pathology–associated changes in PS19 female mice. All data are presented as mean ± SEM. ∗*p* < 0.05, ∗∗*p* < 0.01, and ∗∗∗*p* < 0.001 by unpaired two-tailed *t* test.

To validate the new signaling modules for tauopathy, we selected several key target genes from each signaling pathway and conducted qRT-PCR on these target genes to compare their expression in 4-month-old and 7-month-old PS19 mice. Figure 6*A* shows the expression of genes involved in the dopamine receptor signaling pathway in different brain regions. In the Tha, *Drd1* and *Drd2* levels were significantly increased in 7-month-old PS19 mice. In contrast, *Drd2* and *Adcy* levels were significantly decreased in the OB, and *Drd3* expression was reduced in the Ctx. Interestingly, in the CB, *Drd1*, *Drd2*, and *Drd3* levels were all significantly downregulated. In contrast, *Adcy* expression notably increased in both the SN and CB. Among these changes, the decreased expression of *Drd2* and *Adcy* in the OB, increased *Drd2* expression in the Tha, and decreased *Drd3* expression in the Ctx were consistent with the increased or decreased patterns observed in the corresponding signaling pathways that were discovered using integrative analysis (Supplemental Fig. S4). In the DA-degradation signaling pathway, *Comt* expression was substantially decreased in the OB, in accordance with tau pathology (Fig. 6*B* and Supplemental Fig. S5). The levels of *Maoa* and *Maob*, which are involved in both the DA and 5-HT degradation pathways, were substantially elevated as the tau pathology progressed (Fig. 6*B*, Supplemental Figs. S5, and S7).

**Fig. 7:**
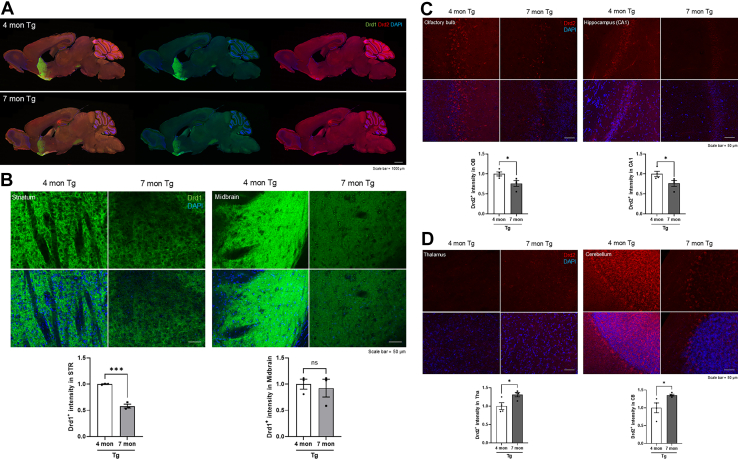
**Regional expression patterns of dopamine receptor in PS19 mice brain.***A*, overview images of whole-brain sections from 4- and 7-month-old PS19 female mice that were immunostained for Drd1 (*green*) and Drd2 (*red*). Scale bar represents 1000 μm. *B*, representative images of Drd1 expression in the STR and midbrain regions, captured via digital zoom. Scale bar represents 50 μm. *C*, representative images showing Drd2 expression in the OB and CA1 region. Scale bar represents 50 μm. *D*, representative images of Drd2 expression in the Tha and CB. Scale bar represents 50 μm. All data are presented as mean ± SEM. ∗*p* < 0.05 and ∗∗∗*p* < 0.001 in the unpaired two-tailed *t* test.

In the 5-HT receptor signaling pathway, *Htr1a* and *Arrb1* expression was significantly increased in the Tha, whereas *Arrb1* expression was significantly decreased in the OB (Fig. 6*C*). Among these changes, only the decrease in *Arrb1* expression in the OB was consistent with proteomic analysis (Supplemental Fig. S6). In the 5-HT degradation signaling pathway, *Aldh2* expression was significantly elevated in the CB, which aligned with the changes that were observed in the integrative proteomic analysis (Fig. 6*D* and Supplemental Fig. S7). To validate the molecular signatures identified by proteomic analysis at the mRNA level, we performed immunohistochemistry to confirm these changes at the protein level. We used Drd1 and Drd2 antibodies to observe DA receptor signaling at the protein level. In the whole-brain overview images, Drd1 expression was predominantly observed along the nigrostriatal dopamine pathway, whereas Drd2 expression showed a relatively widespread distribution across multiple brain regions (Fig. 7*A*). At higher magnification, the Drd1-positive signal was significantly reduced in the STR and showed a decreasing trend in the midbrain, although this was not statistically significant (Fig. 7*B* and Supplemental Fig. S8, *B* and *E*). In contrast, Drd2 expression was significantly decreased in the OB and hippocampal CA1 region (Fig. 7*C* and Supplemental Fig. S8, *A* and *C*), whereas it was significantly increased in Tha and CB (Fig. 7*D* and Supplemental Fig. S8, *D* and *F*).

These changes were consistent with the corresponding alterations observed in proteomic analysis (Supplemental Fig. S4, *A*–*C*). No significant difference in Drd2 expression was observed in the Ctx (Supplemental Fig. S9*D*). These findings indicated that the DA receptor–related molecules in PS19 mice closely aligned with the results of IPA.

## Discussion

In this study, we profiled and integrated proteomes and neurotransmitters in the seven brain regions of mice with progressive tauopathy. Using proteomic analysis, we identified several canonical pathways, including those associated with metabolic abnormalities. We found that six neurotransmitters—DA, 5-HT, HVA, NE, 3-methoxytyramine hydrochloride, and DOPAC—were significantly altered during the progression of tauopathy. Finally, we found that DA- and 5-HT–related signaling modules in the proteomes were closely associated with neurotransmitter alterations, particularly in the Hip and CB, during tauopathy progression. To further explore the potential functional consequences of these molecular changes, we examined whether tauopathy-related neurotransmitter imbalances were linked to neuronal dysfunction. Our region-wide proteomic analysis revealed consistent downregulation of the synaptogenesis signaling pathway across all seven brain regions in 7-month-old PS19 mice (Fig. 3*C*). This global synaptic deactivation supports the notion that tau accumulation may broadly impair synaptic function, thereby contributing to altered neuronal activity. Moreover, to validate the relationship between tauopathy and neurotransmitter changes, we performed histological analyses that focused on the regions of origin of the respective neurotransmitter systems. In both the SN and locus coeruleus, we observed marked accumulation of phosphorylated tau (Supplemental Fig. S10*A*). Notably, in the raphe nucleus, phosphorylated tau colocalized with serotonergic neurons (Supplemental Fig. S10*B*), which further supported the link between tau pathology and neurotransmitter system remodeling that was observed in our integrative proteomic analysis.

DA receptor signals are involved in various neurological processes, such as learning, motivation, and thought processes (32). In DA receptor signaling pathways, Drd2 and Drd3, members of the D2-type receptors, undertake an inhibitory function to reduce neurotransmitter release and suppress neuronal hyperactivity (33, 34). Drd2 is involved in learning, memory, and emotional regulation (35, 36). DRD2 declines with aging (37), which may contribute to the exacerbation of neurodegenerative diseases, including AD. For example, a network meta-analysis reported that, compared to healthy controls, patients with AD had significantly reduced DRD2 levels (15). Moreover, DRD2 homozygotes in patients were closely associated with an increased incidence of psychosis (38). Drd3 is predominantly expressed in regions related to psychotic symptoms and motor function (39, 40), and the expression of DRD3 severely decreased in patients with AD (41). In our study, the Hip, STR, and OB of 7-month-old PS19 mice showed an increase in DA (Fig. 4*B*), whereas Drd1 and Drd2 levels were significantly reduced in each region (Fig. 6A, Supplemental Figs. S4*C*, S9, *A* and *B*). In contrast, in the Tha and CB, DA levels tended to decrease (Fig. 4*B*), whereas IPA, qRT-PCR, and IHC results showed increased Drd2 levels in these regions (Figs. 6*A*, 7, *A*, *C* and *D*, and Supplemental Fig. S8*C*). Moreover, mRNA expression of *Drd3* was significantly decreased in the Ctx of 7-month-old PS19 mice (Fig. 6*A*), which is consistent with previous findings (Fig. 5*A* and Supplemental Fig. S5*A*). The differences in Drd2 expression may be due to the Tha and CB being typically affected during the late stages of AD pathology, according to Braak staging (42). These results may be influenced by the combined effects of early-stage to mid-stage tau pathology and compensatory responses to damage that have occurred in other brain regions in PS19 mice. Accordingly, such stage-specific adaptations may account for the unexpected or regionally divergent outcomes observed. Next, we examined Adcy which transmits inhibitory signals from DA receptors by producing cyclic-3′,5′-adenosine monophosphate (43). ADCY activity significantly declined in the AD group (44, 45, 46). In our study, the mRNA expression of *Adcy* decreased in 7-month-old PS19 mice (Fig. 6*A*), which reflected a reduction in signaling (Fig. 5*A* and Supplemental Fig. S5*C*). However, studies on the association between Adcy and AD are limited, and recent investigations are relatively scarce.

We examined the expression of MAO, which mediates DA and 5-HT degradation. MAO has two isoforms, Maoa and Maob, encoded by separate genes (47). Maoa abnormalities cause monoaminergic and mitochondrial dysfunction, which affect neuronal survival in AD (48, 49). Maob is enhanced in reactive astrocytes surrounded by amyloid-β and presents higher expression in the AD postmortem brain (50). Both Maoa and Maob levels are elevated in AD brain tissue (51, 52). Similarly, we observed upregulated DA and 5-HT degradation (Fig. 4, *B* and *D*, Supplemental Figs. S5*B* and S7) and an increase in the expression of *Maoa* and *Maob* in 7-month-old PS19 mice (Fig. 6*B*), which indicated the acceleration of DA and 5-HT degradation via MAO as AD progressed. As a downstream mediator, Aldh catalyzes the oxidative deamination of 5-hydroxyindole acetate (53). Furthermore, with the onset of AD, ALDH expression and its activity level are elevated (54, 55). Consistently, the expression of *Aldh2* was elevated in 7-month-old PS19 mice (Fig. 6*D*). Our study investigated MAO-mediated signal changes during AD progression, whereas previous studies have focused on comparing patients with AD and healthy controls (56). Thus, we suggest that increased MAO signaling contributes to the onset and progression of AD. Unlike the MAO pathway, another mediator, COMT, is involved in the degradation of DA (57). COMT mediates the degradation of DA and the conversion of DA to 3-methoxytyramine (58) and is involved in alterations in cognition, emotion, and personality (59). We observed that the *Comt* level declined in the OB with tauopathy (Fig. 6*B*), and this was accompanied by a reduction in DA degradation via COMT (Fig. 5*A* and Supplemental Fig. S5*A*). However, few studies have investigated the direct relationship between COMT expression and AD.

In addition to the evaluation of DA signaling modules, we conducted comprehensive studies on the signal pathways related to 5-HT. β-arrestin acts as scaffold for the ERK1/2 and other cascades and facilitates G protein-coupled receptor-mediated MAP kinase activation in 5-HT receptor signaling (60). Ubiquitously expressed in mammalian tissues (61), *ARRB1* is significantly decreased in the serum of patients with AD, and this ARRB1 reduction decreased ERK activity downstream, which enhanced the cell death induced by Aβ25 to 35. Similar to a previous study, *Arrb1* expression was reduced in the 7-month-old PS19 mice (Fig. 6*C*), which aligned with a decrease in 5-HT signaling as tauopathy progressed (Fig. 4*B* and Supplemental Fig. S6).

In addition, we noted that the proteomic changes were not always recapitulated at the transcript level. This incomplete concordance is not unexpected and may reflect post-transcriptional regulatory mechanisms, including altered translational control and protein stability/turnover. Because the transcriptomic and proteomic datasets were generated using different experimental platforms and statistical frameworks, we assessed mRNA–protein concordance qualitatively based on the direction of statistically significant changes rather than by directly comparing effect-size magnitudes. Using this approach, we found that many target–region pairs showed significant protein-level changes in the absence of significant mRNA changes, which suggests that post-transcriptional regulation contributes to the region-specific signaling alterations that are observed in PS19 mice. In contrast, a subset of targets exhibited concordant directional changes at both the mRNA and protein levels, indicating that transcriptional regulation may also contribute to specific regions. The target-by-target concordance assessment is summarized in Supplemental Table S6.

There are many reports on multiomics that integrate information from genomics and proteomics (62, 63) and thereby provide meaningful insights into neuroscience that are impossible using traditional approaches, such as new molecular targets and brain regions for specific diseases (64, 65). However, there are reports of discrepancies between genomics and proteomics, which may have originated for several reasons, including the majority of signal transduction being performed by proteins, which comprise very diverse modification phenomena, and they are not parallel information but have sequential relationships (66). The integration of the profiles of neurotransmitters and proteomes is as yet unelucidated. Protein enzymes primarily regulate neurotransmitters. Therefore, changes in proteomic profiles are directly linked to neurotransmitter homeostasis (67). Beyond the traditional multiomics approach, this integrated proteomics approach could provide new insights into the understanding of brain diseases and new molecular targets for treating brain diseases. This study identified region-specific signaling modules in the PS19 mouse brain that were synchronized with neurotransmitter changes. These results provide solid information for the study of AD. Additive integration of other omics such as transcriptomics and genomics is necessary to obtain more precise and relevant information (68). To handle such large-scale data, artificial intelligence must be extensively applied (69). Therefore, this approach may be the only option for future brain studies.

## Data Availability

The mass spectrometry proteomics data have been deposited to the ProteomeXchange Consortium via the PRIDE partner repository with the dataset identifier PXD066515 and are currently accessible to reviewers.

## Supplemental Data

This article contains supplemental data.

## Conflict of interests

The authors declare no competing interests.
