# Supplementary material for: An Integrative Proteomic Approach to Reveal Altered Signaling Modules During Alzheimer’s Disease Progression in PS19 Tauopathy Mice
Source: Mol Cell Proteomics. 2026 May 5;25(6):101580. doi: 10.1016/j.mcpro.2026.101580 (PMC13241738; doi:10.1016/j.mcpro.2026.101580)
Supplement: Supplemental Data [file mmc1.docx]

< *Molecular & Cellular Proteomics* >

Supplementary information for

**An integrative proteomic approach to reveal altered signaling modules during Alzheimer’s disease progression in PS19 tauopathy mice**

Eunji Cho^1,2,&^, Seulah Lee^1,&^, Hagyeong Lee^1^, Jaehoon Kim^1^, Yang Woo Kwon^3^, Hyang-Sook Hoe^2^, Dayea Kim^3^ and Jong Hyuk Yoon^1,*^

***Corresponding Author**:

Jong Hyuk Yoon, Ph.D. Neurodegenerative Diseases Research Group, Korea Brain Research Institute, Daegu 41062, Republic of Korea. Tel: +82-53-980-8341; Fax: +82-53-980-8399; E-mail: [jhyoon@kbri.re.kr](mailto:jhyoon@kbri.re.kr), ORCID: 0000-0002-4090-3832

This file includes:

Supplementary Figure 1 to 11.............................................................................. 2

Supplementary Table 1-6....................................................................................17

**Supplementary Figure 1.** Whole blot image corresponding to the cropped western blot shown in Figure 1A


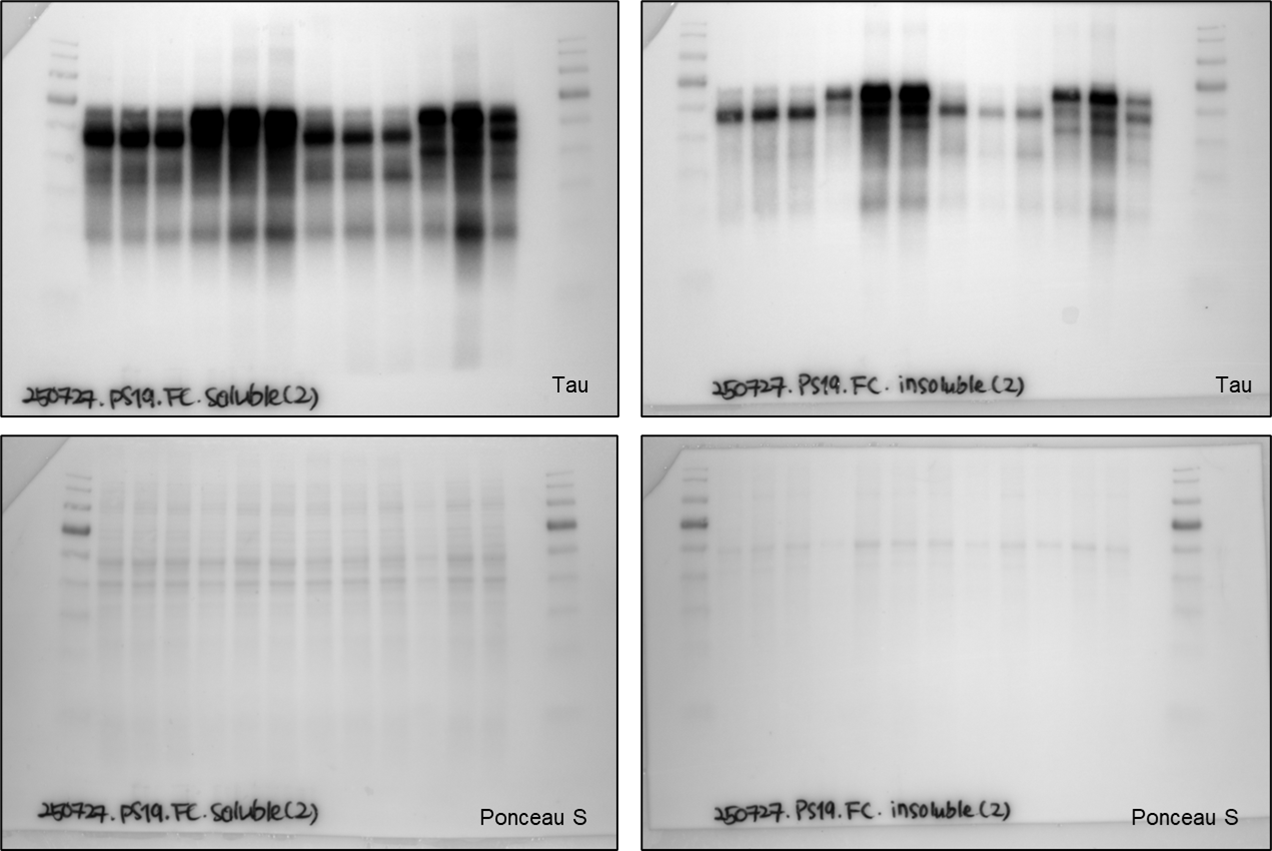


**Supplementary Figure 2.** Assessment of LC–MS/MS run consistency using total ion chromatograms (TICs).

(A) TICs from hippocampus (Hip) samples across experimental groups (4-month WT, 4-month Tg, 7-month WT, and 7-month Tg). (B) TICs from cortex (Ctx) samples across the same experimental groups. (C) TICs of biological replicates from the substantia nigra (SN) of 4-month-old WT mice. (D) TICs of biological replicates from the substantia nigra (SN) of 4-month-old Tg mice. (E) TICs from seven brain regions (olfactory bulb, OB; striatum, STR; hippocampus, Hip; cortex, Ctx; thalamus, Tha; substantia nigra, SN; and cerebellum, CB) obtained from 7-month-old WT mice.

**
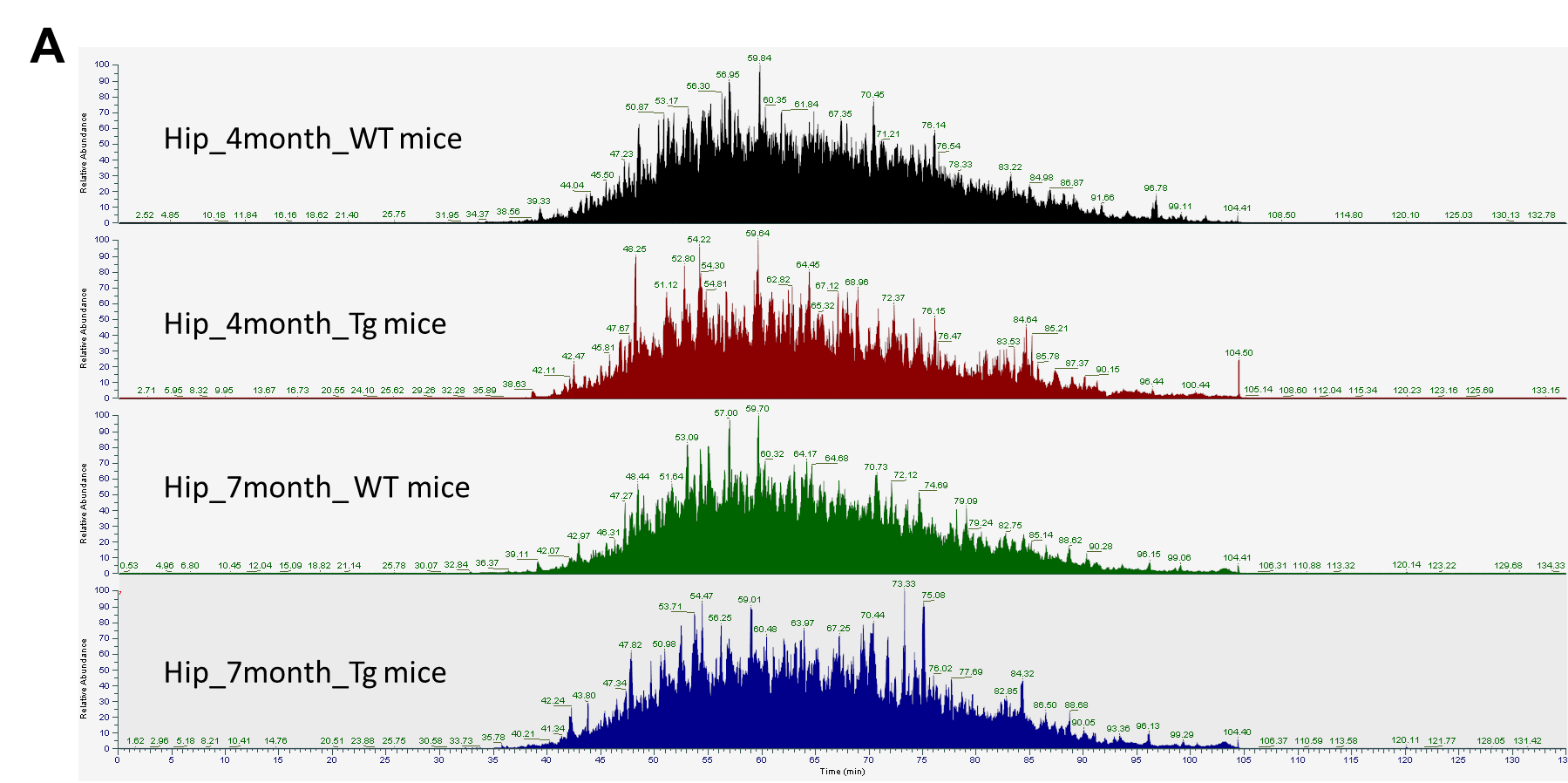
**

**
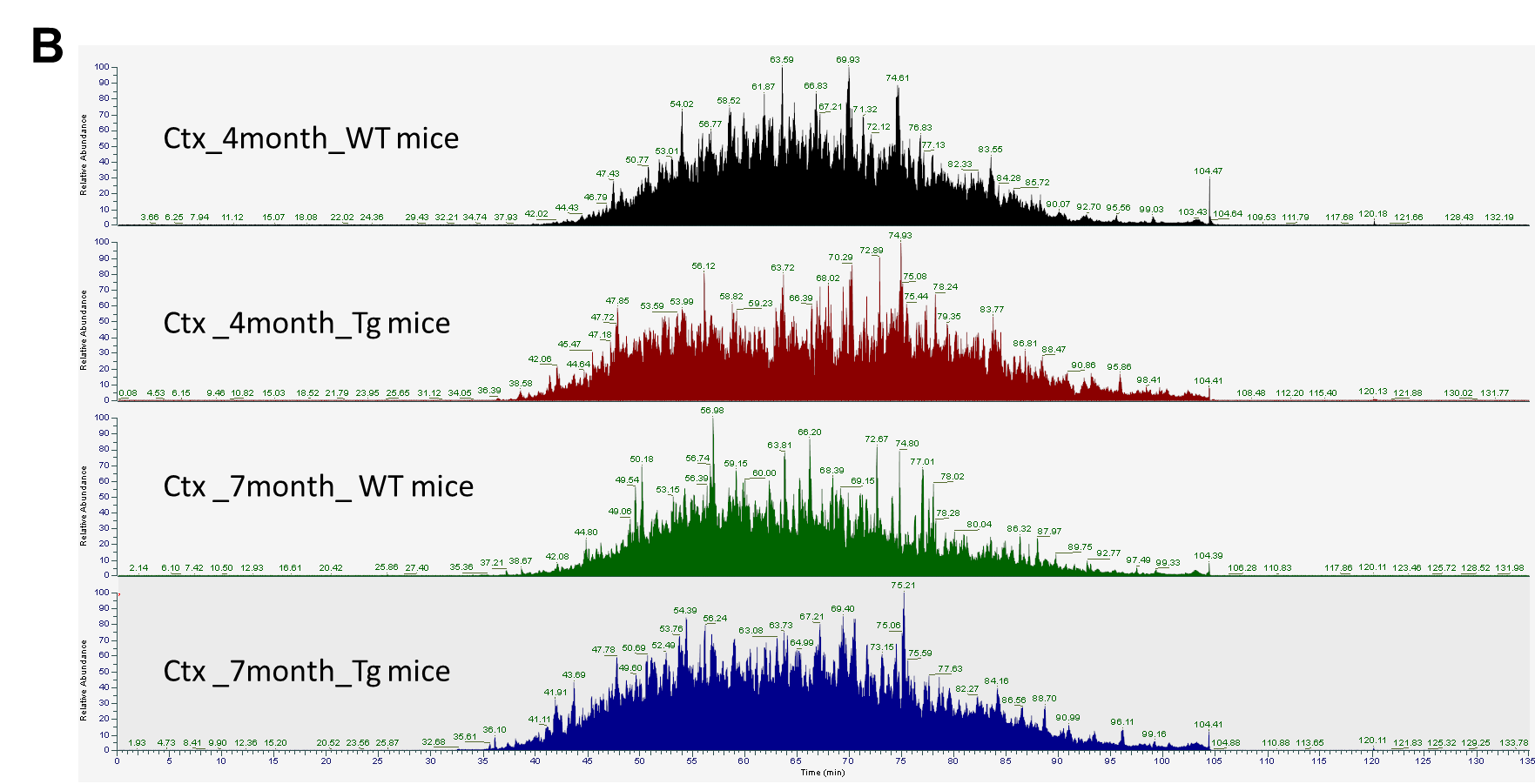
**

**
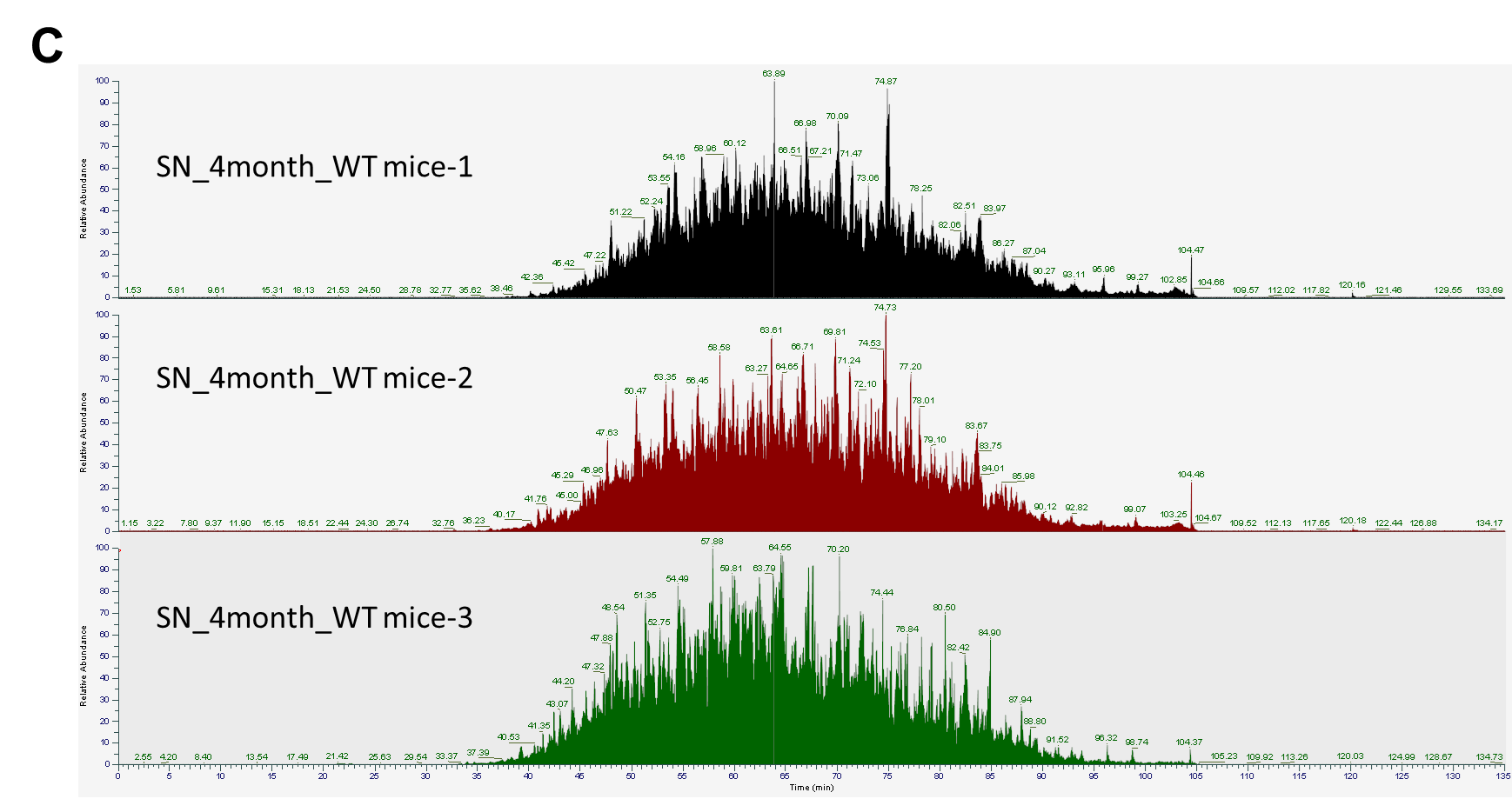
**

**
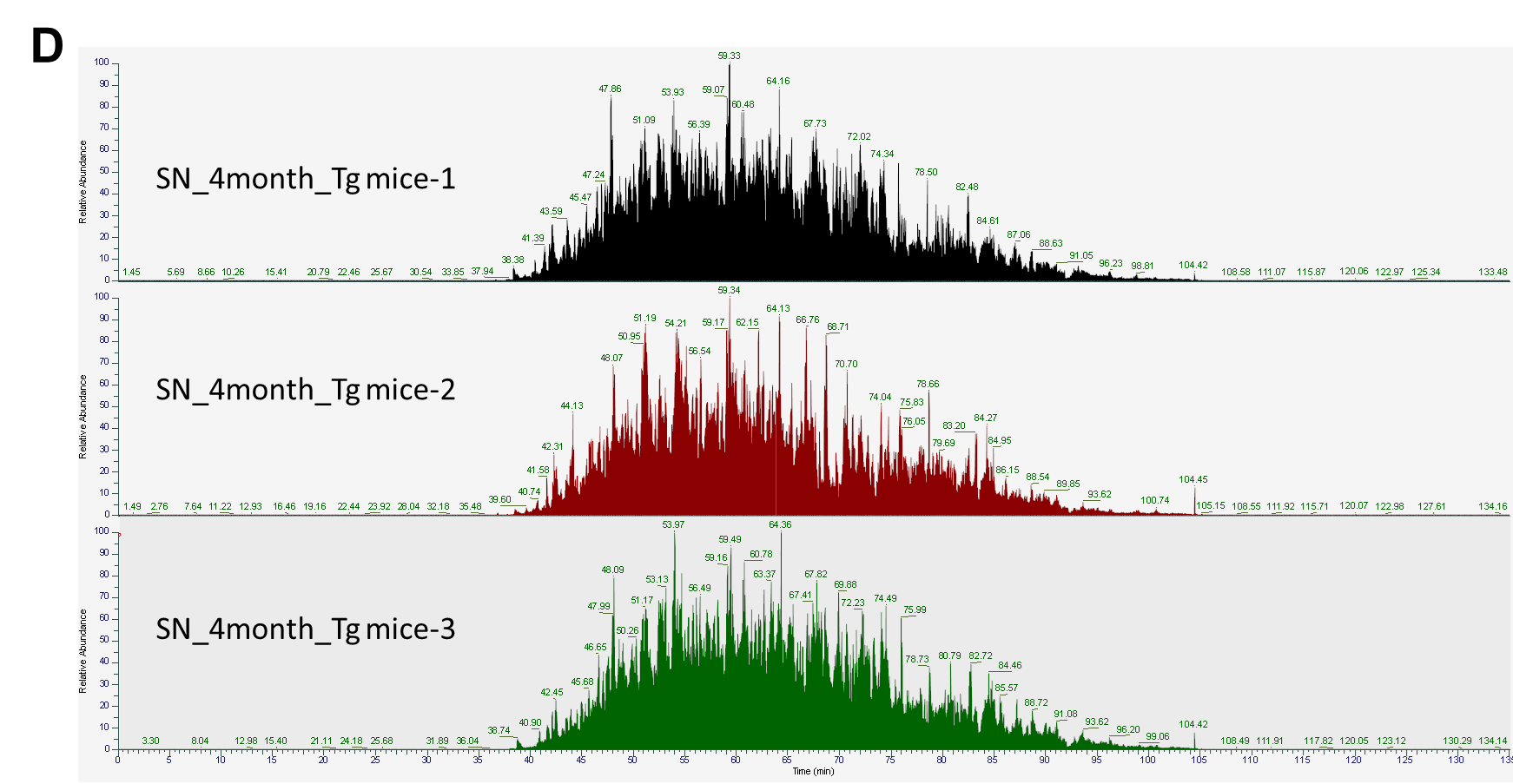
**

**
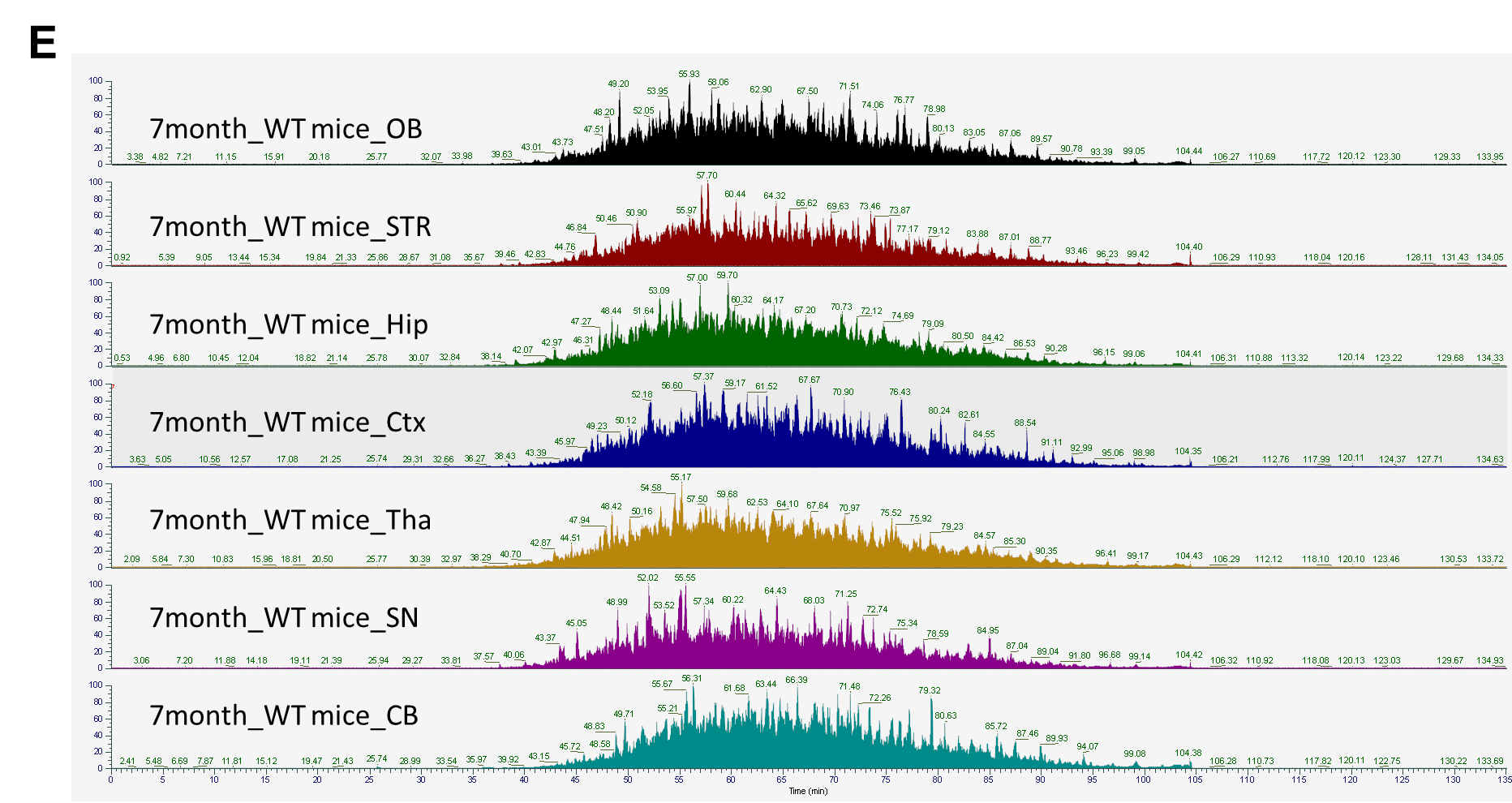
**

**Supplementary Figure 3. Quantitative profiling of seven neurotransmitters across brain regions in 4-month-old and 7-month-old WT and PS19 female mice.** (A) Vanillylmandelic acid (VMA), (B) Tyrosine (TYR), (C) Norepinephrine (NE), (D) Epinephrine (EPI), (E) 3-Methoxytyramine (3-MT), (F) Tryptophan (TRP), and (G) Homovanillic acid (HVA) levels were measured in seven brain regions—olfactory bulb (OB), striatum (STR), hippocampus (Hip), cortex (Ctx), thalamus (Tha), substantia nigra (SN), and cerebellum (CB)—of WT and PS19 mice at the 4 months and 7 months of age. Data are presented as mean ± SEM. Statistical significance was determined using two-way ANOVA followed by Sidak's test. **p* < 0.05, ***p* < 0.01, ****p* < 0.001, and *****p* < 0.0001. Each group comprised *n* = 3 female mice.


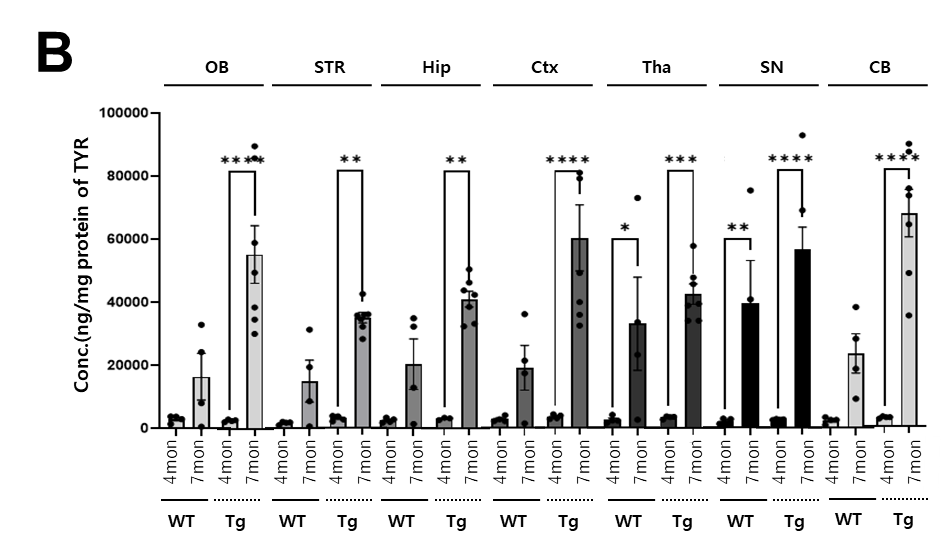

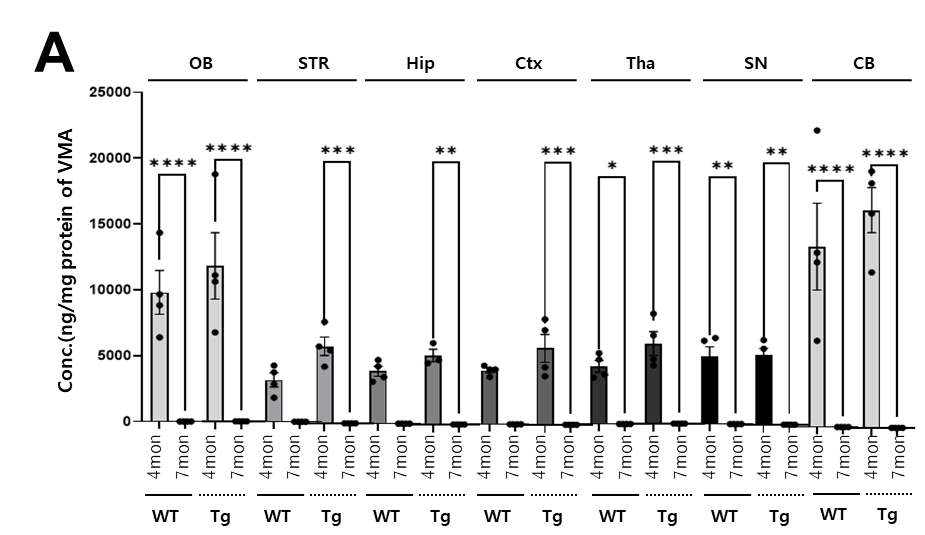


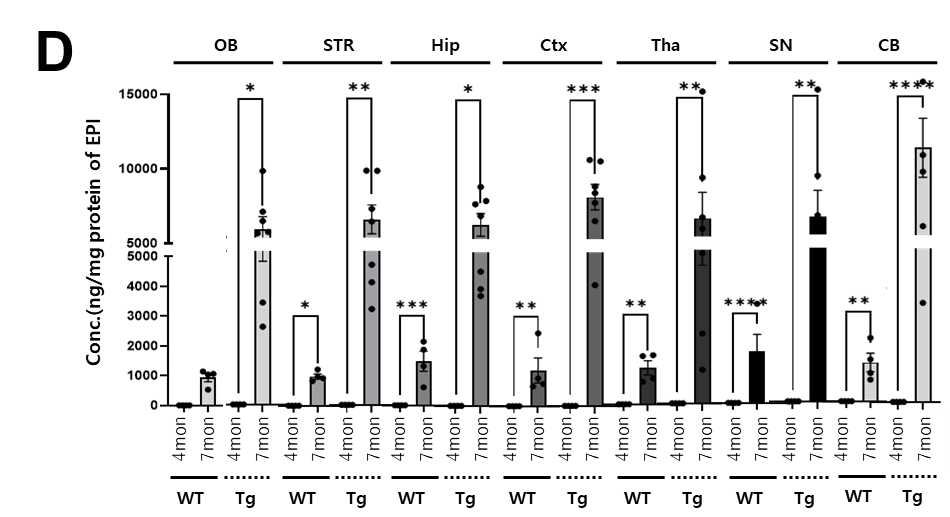

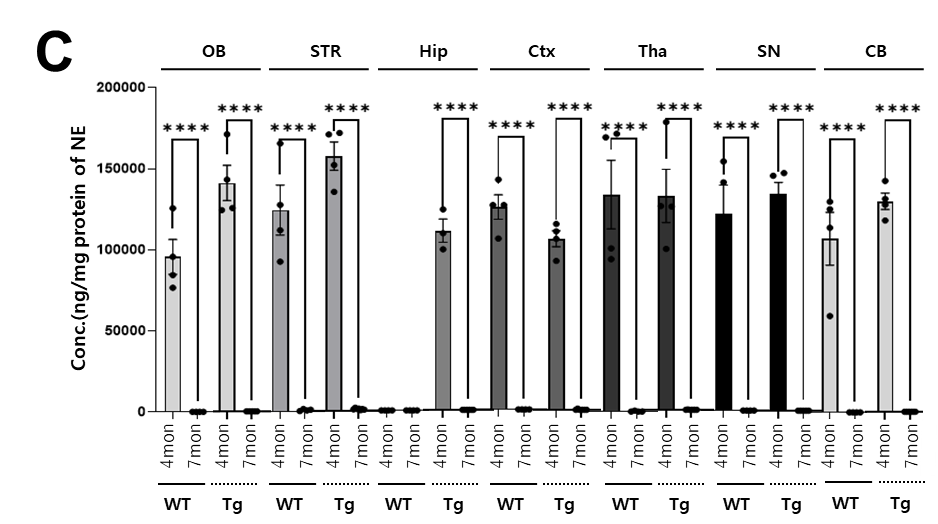


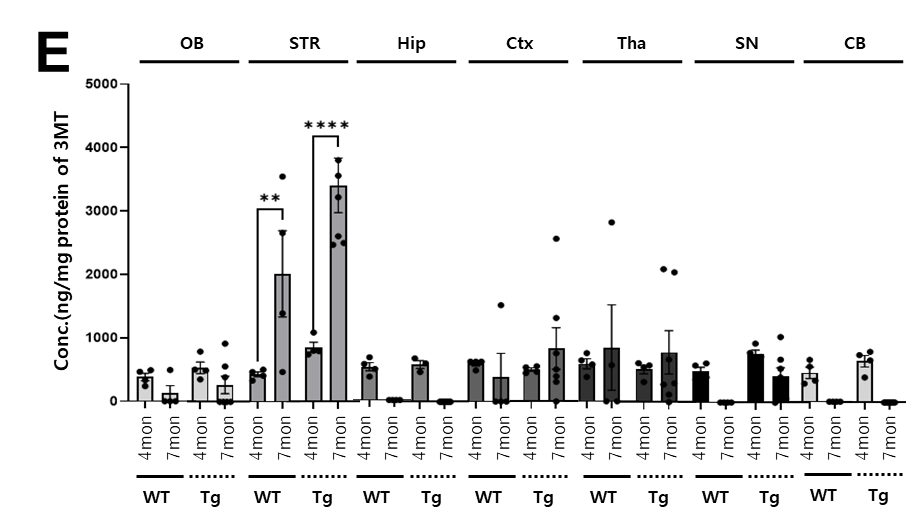


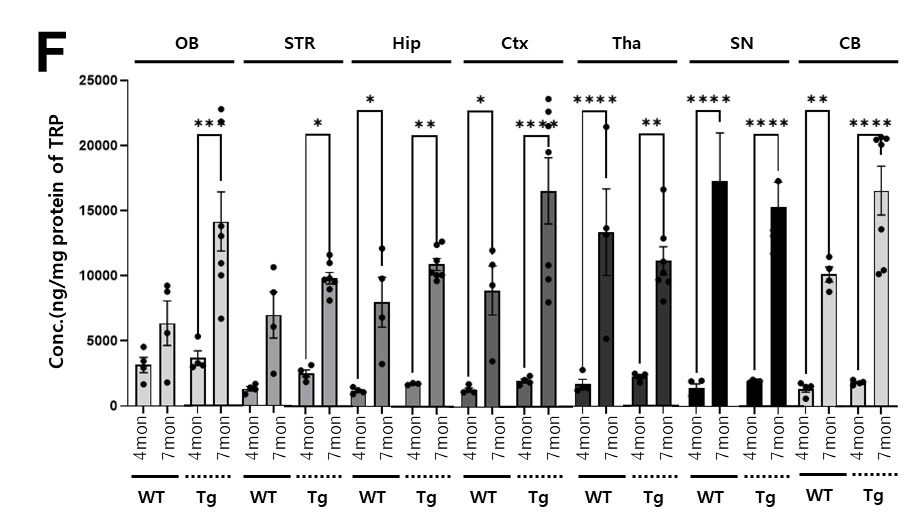


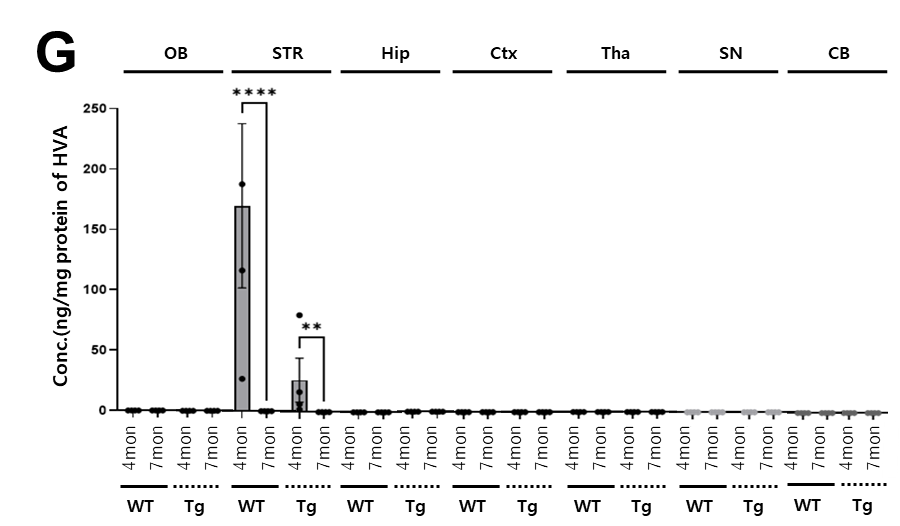


**Supplementary Figure 4.** Validation of the target genes of the discovered dopamine receptor-signaling modules

**
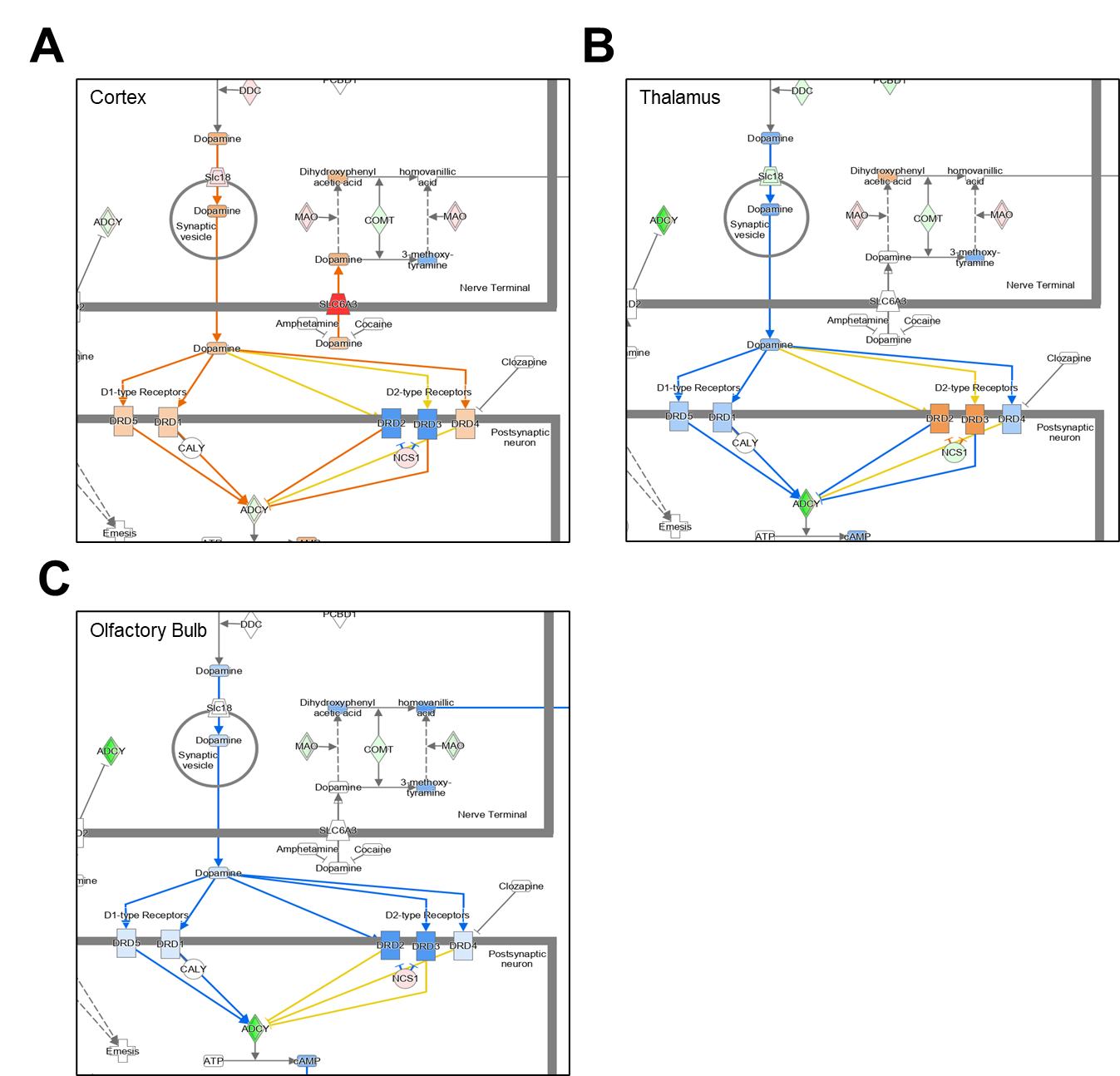
**

**Supplementary Figure 5.** Validation of the target genes of the discovered dopamine degradation-signaling modules

**
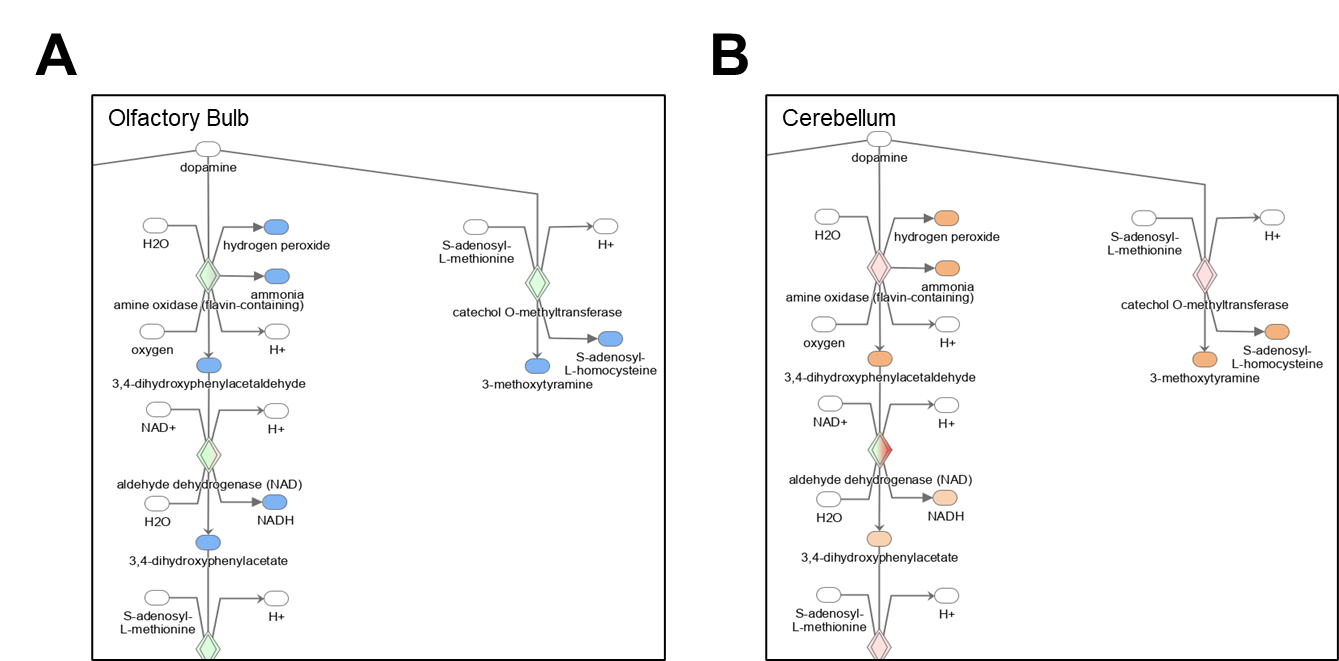
**

**Supplementary Figure S6.** Validation of the target genes of the discovered serotonin receptor-signaling modules.

**
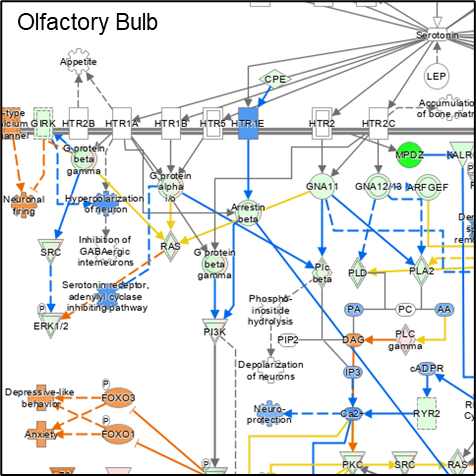
**

**Supplementary Figure 7.** Validation of target genes of the discovered serotonin degradation-signaling modules.


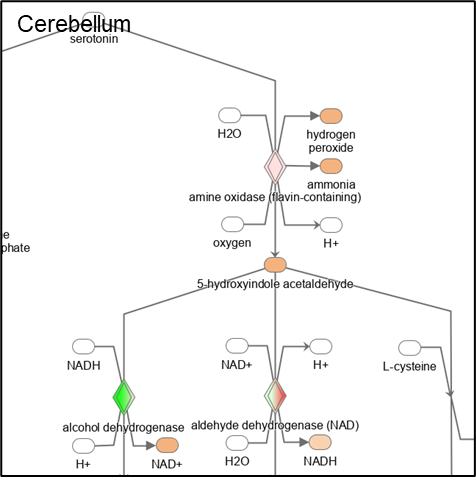


**Supplementary Figure 8. Low-magnification overview images of brain regions shown in Figure 6.** (A–F) Representative low-magnification images corresponding to regions presented in Figure 6, showing Drd1 (green) and Drd2 (red) immunofluorescence staining across various brain areas, including the OB (A), STR (B), Hip (C), Tha (D), midbrain (E), and CB (F). These images serve as the original field views from which the higher-magnification images in Figure 6 were selected. Scale bar = 100 µm.


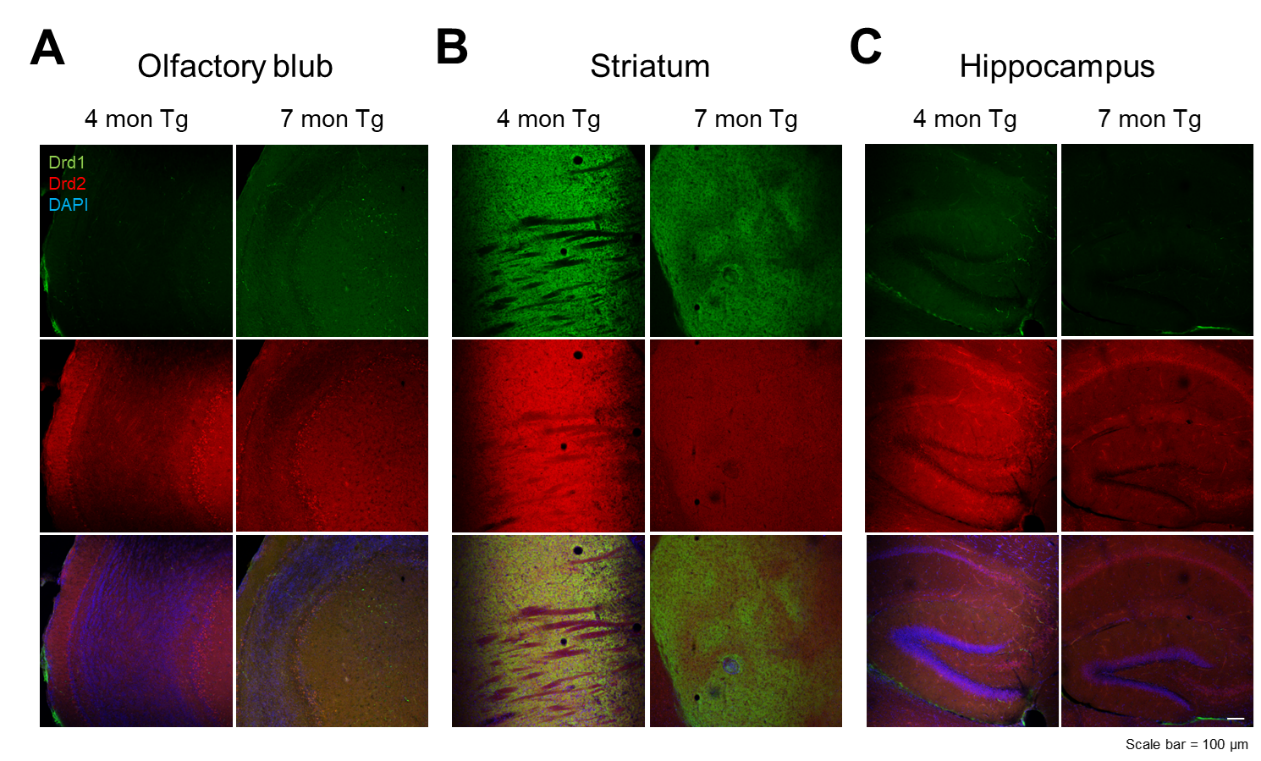


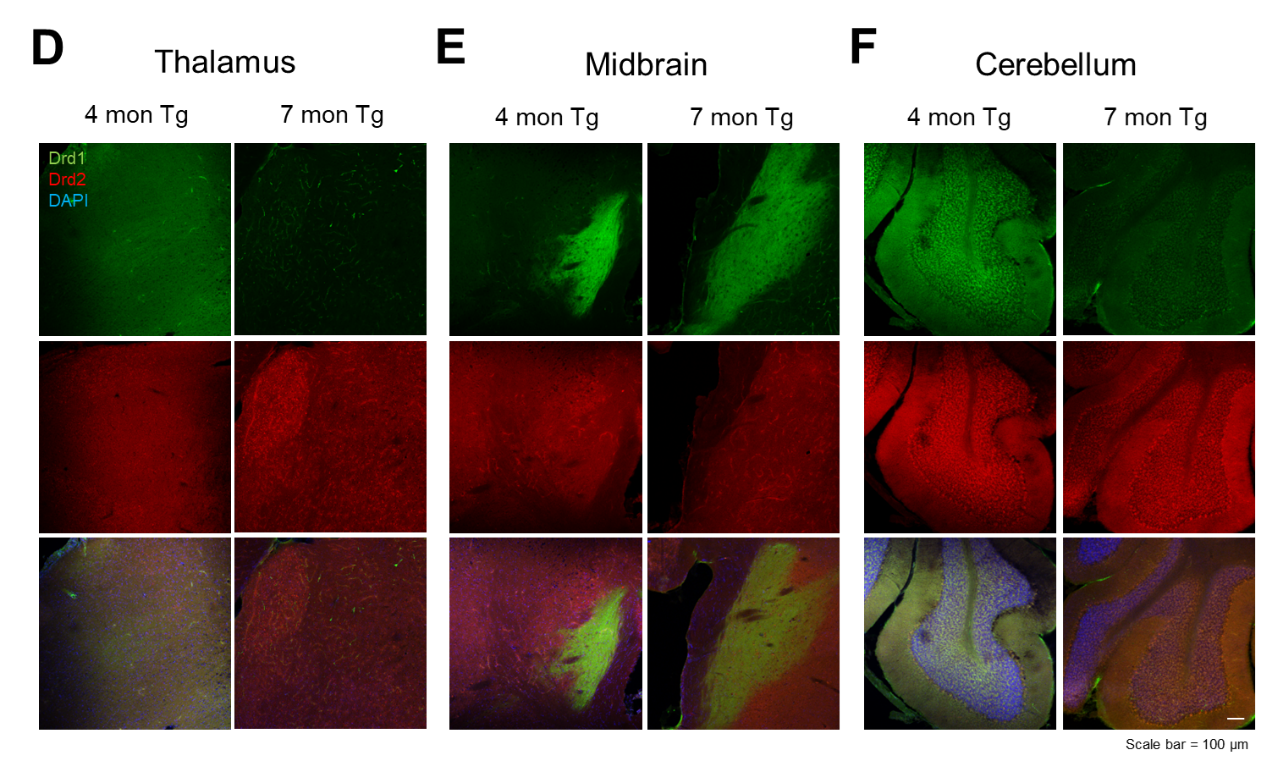


**Supplementary Figure 9.** Validation of the target proteins of the discovered dopamine receptor-signaling modules.
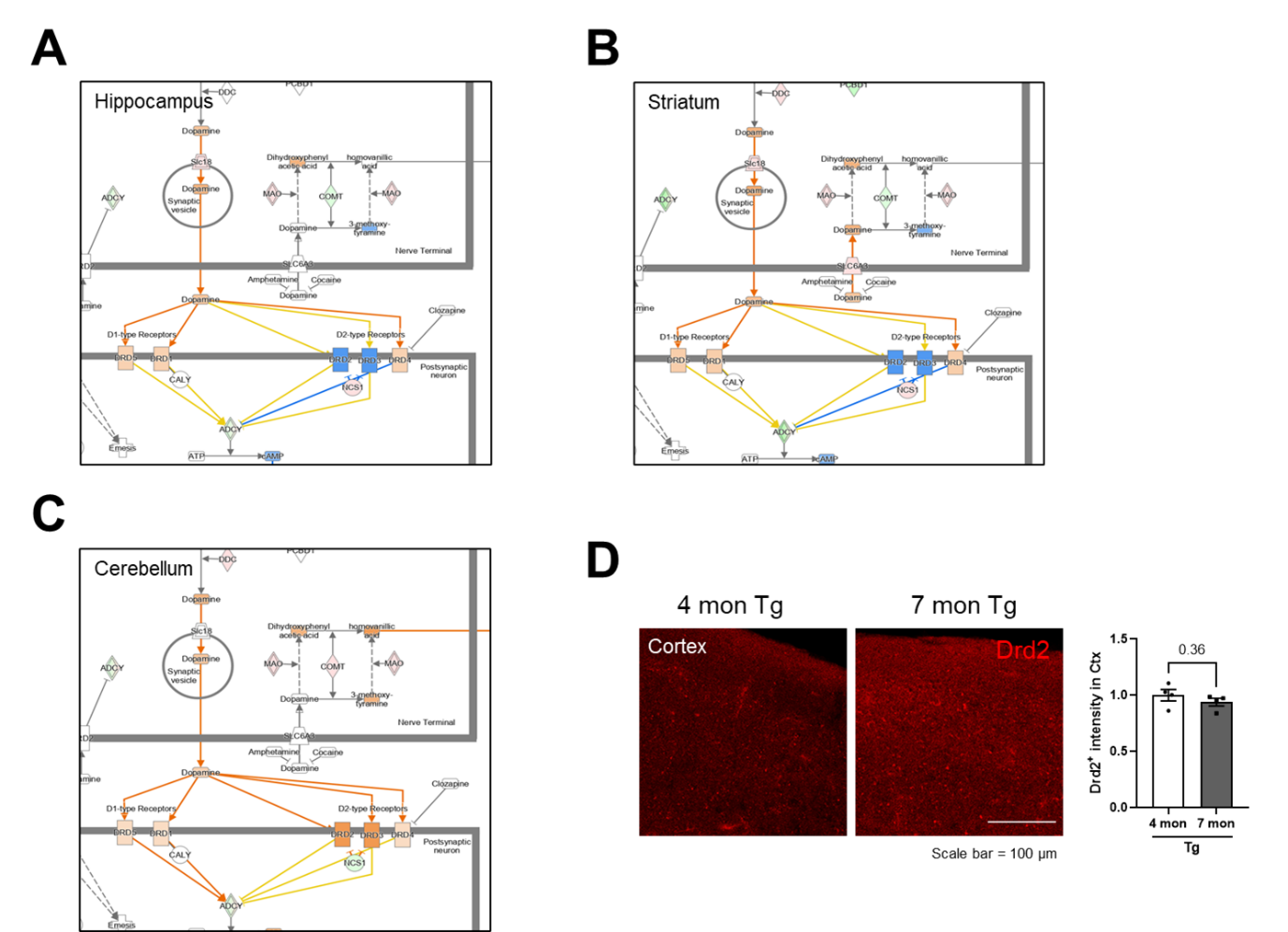


**Supplementary Figure 10. Tau pathology in neurotransmitter-producing regions supports proteomic findings.** (A) Representative images showing co-localization of phosphorylated tau (AT8) with TH-positive neurons in the SN and LC. Scale bar = 20 μm. (B) Representative images showing co-localization of AT8 with TPH2-positive serotonergic neurons in the RN. Scale bar = 20 μm. Data are presented as mean ± SEM. Statistical analysis was performed using one-way ANOVA followed by Bonferroni’s post hoc test. **p* <0.05; *n* = 3–4 per group.


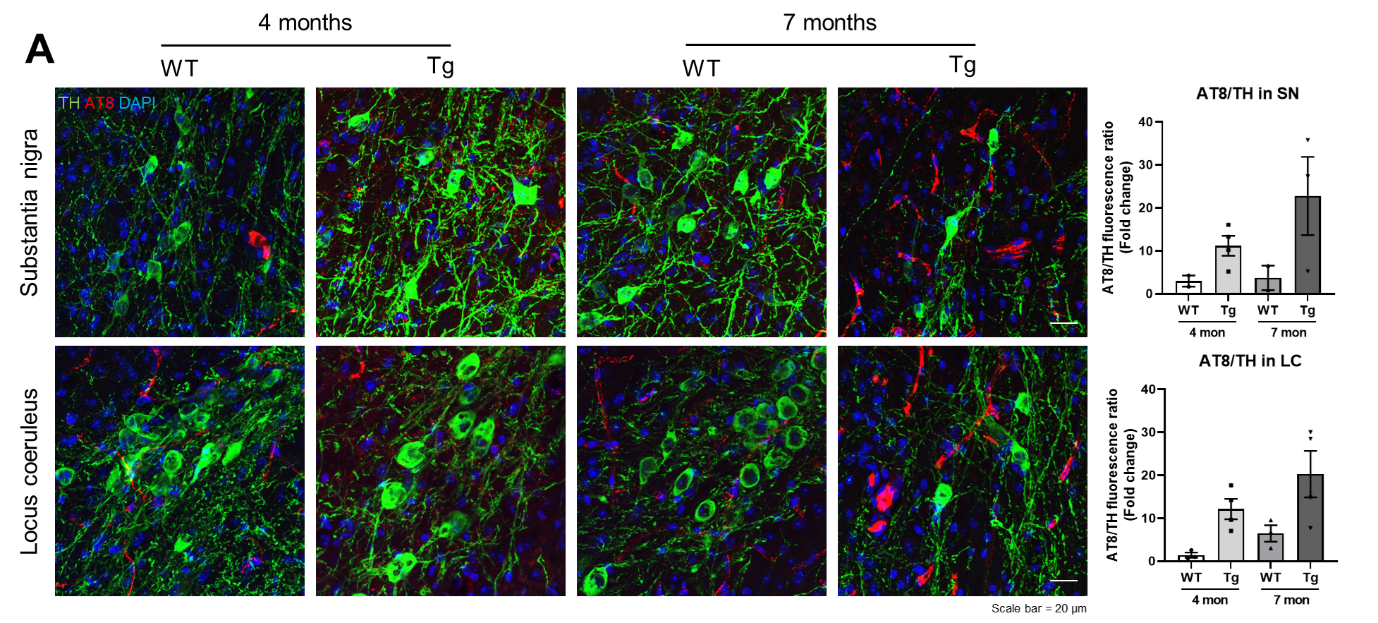


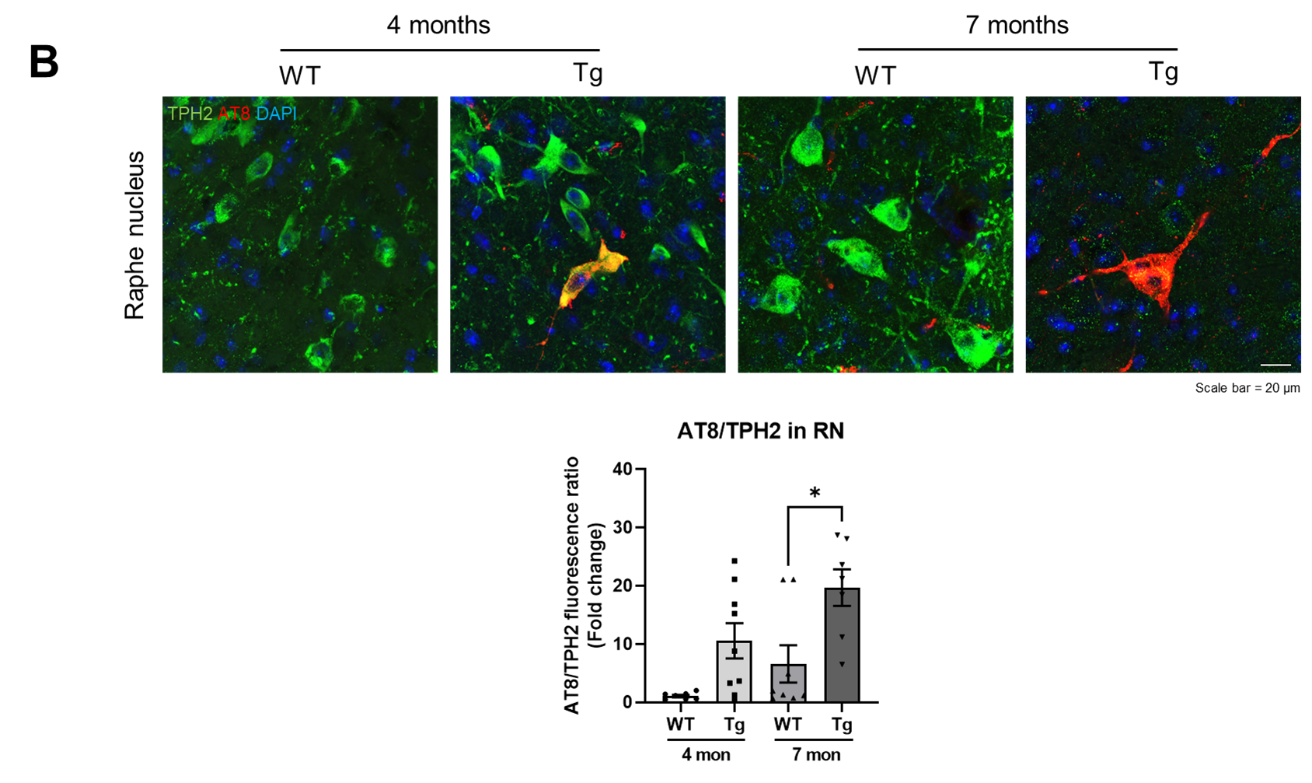


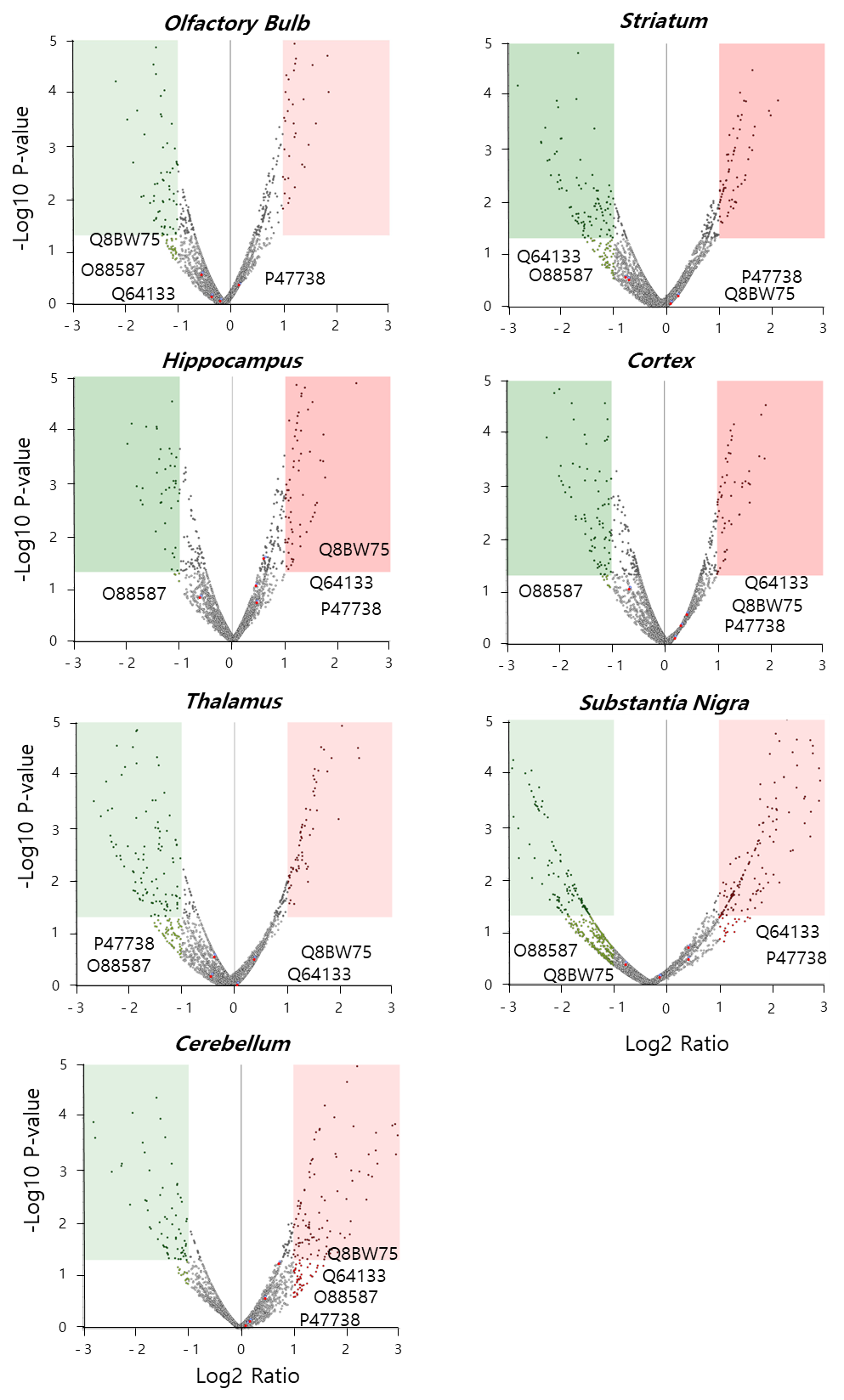
**Supplementary Figure 11. Summary of candidate proteins selected for downstream validation.** Candidate proteins were identified through integrative analyses that combined quantitative proteomic changes and pathway-level insights derived from GSEA and IPA. The proteins shown correspond to those that were further examined and experimentally validated.

**Supplementary Table 1. Primary antibodies used for immunostaining**

| **Target** | **Host** | **Company** | **Catalog No.** | **Dilution** |
| --- | --- | --- | --- | --- |
| AT8 (pTau Ser202/Thr205) | Mouse | Thermo Fisher | MN1020 | 1:1000 |
| Drd1 (Dopamine receptor D1) | Rat | Millipore | D2944 | 1:500 |
| Drd2 (Dopamine receptor D2) | Rabbit | Millipore | AB5084P | 1:500 |
| TH (Tyrosine hydroxylase) | Rabbit | Millipore | AB152 | 1:1000 |
| TPH2 (Tryptophan hydroxylase 2) | Rabbit | Novus | NB100-74555 | 1:1000 |

This table lists the primary antibodies used for double immunofluorescence staining, including target antigens, host species, dilution factors, and commercial sources.

**Supplementary Table 2. Order of sample analysis for LC–MS/MS–based proteomics and HPLC-based neurotransmitter measurements**

| **Sample ID** | **Age** | **Group** | **Proteomics run order** | **HPLC**  **neurotransmitter run order** |
| --- | --- | --- | --- | --- |
| 32-OB, STR, Hip, Ctx, Tha, SN, CB | 7mon | WT | 78-94 | 1-7 |
| 226-OB, STR, Hip, Ctx, Tha, SN, CB | 7mon | WT | 95-101 | 8-14 |
| 259-OB, STR, Hip, Ctx, Tha, SN, CB | 7mon | WT | 102-108 | 15-21 |
| 260-OB, STR, Hip, Ctx, Tha, SN, CB | 7mon | WT | 109-115 | 22-28 |
| 35-OB, STR, Hip, Ctx, Tha, SN, CB | 7mon | Tg | 116-122 | 29-35 |
| 229-OB, STR, Hip, Ctx, Tha, SN, CB | 7mon | Tg | 123-129 | 36-42 |
| 228-OB, STR, Hip, Ctx, Tha, SN, CB | 7mon | Tg | 130-136 | 43-49 |
| 215-OB, STR, Hip, Ctx, Tha, SN, CB | 7mon | Tg | 137-143 | 50-56 |
| 216-OB, STR, Hip, Ctx, Tha, SN, CB | 7mon | Tg | 144-150 | 57-63 |
| 241-OB, STR, Hip, Ctx, Tha, SN, CB | 7mon | Tg | 151-157 | 64-70 |
| 2300-OB, STR, Hip, Ctx, Tha, SN, CB | 7mon | Tg | 158-164 | 71-77 |
| 729-OB, STR, Hip, Ctx, Tha, SN, CB | 4mon | WT | 165-171 | 221-227 |
| 701-OB, STR, Hip, Ctx, Tha, SN, CB | 4mon | WT | 172-178 | 228-234 |
| 706-OB, STR, Hip, Ctx, Tha, SN, CB | 4mon | WT | 179-185 | 235-241 |
| 780-OB, STR, Hip, Ctx, Tha, SN, CB | 4mon | WT | 186-192 | 242-248 |
| 756-OB, STR, Hip, Ctx, Tha, SN, CB | 4mon | Tg | 193-199 | 249-255 |
| 753-OB, STR, Hip, Ctx, Tha, SN, CB | 4mon | Tg | 200-206 | 256-262 |
| 549-OB, STR, Hip, Ctx, Tha, SN, CB | 4mon | Tg | 207-213 | 263-269 |
| 548-OB, STR, Hip, Ctx, Tha, SN, CB | 4mon | Tg | 214-220 | 270-276 |

This table summarizes the randomized order in which samples were analyzed to assess and minimize potential run-order effects.

**Supplementary Table 3. List of primers for targeted genes to validate the selected signaling modules**

| **Gene name** | **Forward primer**  **(5’ to 3’)** | **Reverse primer**  **(5’ to 3’)** |
| --- | --- | --- |
| Adenylate cyclase 1 (*Adcy1*) | GTCGGATGGATAGCACTGGG | TTCACGCTGACTTTGCCTCT |
| Aldehyde dehydrogenase 2 (*Aldh2*) | TGGTCCTGAAATGTCTCCGC | CAGGCTCATGGCGGGTATAG |
| Arrestin beta 1 (*Arrb1*) | ACCCAGCTCAACATTCTGCA | TGGAAGCCAGATTCGTGTCC |
| Catechol-O-methyltransferase (*Comt*) | GCTGCTGTCTCATTGGGTCT | CTGCTGCACGAACTCAAACC |
| Dopamine receptor D1 (*Drd1*) | AGGTTGAGCAGGACATACGC | TTGCTTCTGGGCAATCCTGT |
| Dopamine receptor D2 (*Drd2*) | CTACATCGTTCTCCGCAAGC | CAGCATCCTTGAGTGGTGTC |
| Dopamine receptor D3 (*Drd3*) | CCCTACAGACCACCACCAAC | CCTCCAAGTACACCACCCAC |
| 5-hydroxytryptamine receptor 1A (*Htr1a*) | CTCATGGTGTCAGTGCTGGT | CCAGGGCGATAAACAGGTCA |
| Monoamine oxidase A (*Maoa*) | GGCTACATGGAAGGTGCAGT | GGACTCAGGCTCTTGAACCC |
| Monoamine oxidase B (*Maob*) | CTGCAGCCCGTCCATTATGA | GGCTGACGTAGAACCCTTCC |

This table includes the primer sequences used for the quantitative PCR analysis of genes that were selected to validate the identified signaling modules.

**Supplementary Table 4. Detailed protein identification and quantitative information for all identified proteins across seven brain regions in WT and Tg mice during disease progression (4–7 months)**

Separate Excel file

The table is provided as a separate Excel file that contains 14 sheets which correspond to region-wise differential proteomic comparisons. Each sheet includes protein accession numbers, source database (UniProt), number of distinct peptides, sequence coverage (%), and protein-level quantitative measurements from label-free quantification.

**Supplementary Table 5. List of proteins involved in the selected signaling modules related to tauopathy progression in PS19 mice**

Separate Excel file

This table lists proteins that show region-specific changes in dopamine and serotonin signaling pathways from 4 to 7 months in PS19 mice that were identified through integrated proteomic and neurotransmitter analyses.

**Supplementary Table 6. Qualitative concordance between transcriptomic and proteomic changes across targets and PS19 brain regions**

| Target | Region | mRNA | Protein | Concordance |
| --- | --- | --- | --- | --- |
| Drd1 | OB | - | - | No change |
|  | STR | - | Up | Protein-only (PT-regulation likely) |
|  | Hip | - | Up | Protein-only (PT-regulation likely) |
|  | Ctx | - | Up | Protein-only (PT-regulation likely) |
|  | Tha | Up | Down | Discordant |
|  | SN | - | - | No change |
|  | CB | Down | Up | Discordant |
| Drd2 | OB | Down | - | mRNA-only |
|  | STR | - | Down | Protein-only (PT-regulation likely) |
|  | Hip | - | Down | Protein-only (PT-regulation likely) |
|  | Ctx | - | Down | Protein-only (PT-regulation likely) |
|  | Tha | Up | Up | Concordant |
|  | SN | - | - | No change |
|  | CB | Down | Up | Discordant |
| Drd3 | OB | - | - | No change |
|  | STR | - | Down | Protein-only (PT-regulation likely) |
|  | Hip | - | Down | Protein-only (PT-regulation likely) |
|  | Ctx | Down | Down | Concordant |
|  | Tha | - | Up | Protein-only (PT-regulation likely) |
|  | SN | - | - | No change |
|  | CB | Down | Up | Discordant |
| Adcy | OB | - | Down | Protein-only (PT-regulation likely) |
|  | STR | - | Down | Protein-only (PT-regulation likely) |
|  | Hip | - | Down | Protein-only (PT-regulation likely) |
|  | Ctx | - | Down | Protein-only (PT-regulation likely) |
|  | Tha | - | Down | Protein-only (PT-regulation likely) |
|  | SN | Up | - | mRNA-only |
|  | CB | Up | Down | Discordant |
| Comt | OB | Down | Down | Concordant |
|  | STR | - | Down | Protein-only (PT-regulation likely) |
|  | Hip | - | Down | Protein-only (PT-regulation likely) |
|  | Ctx | - | Down | Protein-only (PT-regulation likely) |
|  | Tha | - | Down | Protein-only (PT-regulation likely) |
|  | SN | - | Down | Protein-only (PT-regulation likely) |
|  | CB | - | Up | Protein-only (PT-regulation likely) |
| Maoa | OB | - | Down | Protein-only (PT-regulation likely) |
|  | STR | - | Up | Protein-only (PT-regulation likely) |
|  | Hip | - | Up | Protein-only (PT-regulation likely) |
|  | Ctx | - | Up | Protein-only (PT-regulation likely) |
|  | Tha | - | Up | Protein-only (PT-regulation likely) |
|  | SN | - | Down | Protein-only (PT-regulation likely) |
|  | CB | Up | Up | Concordant |
| Maob | OB | - | Down | Protein-only (PT-regulation likely) |
|  | STR | - | Up | Protein-only (PT-regulation likely) |
|  | Hip | - | Up | Protein-only (PT-regulation likely) |
|  | Ctx | - | Up | Protein-only (PT-regulation likely) |
|  | Tha | - | Up | Protein-only (PT-regulation likely) |
|  | SN | - | Down | Protein-only (PT-regulation likely) |
|  | CB | Up | Up | Concordant |
| Htr1a | OB | - | - | No change |
|  | STR | - | Up | Protein-only (PT-regulation likely) |
|  | Hip | - | Down | Protein-only (PT-regulation likely) |
|  | Ctx | - | Up | Protein-only (PT-regulation likely) |
|  | Tha | Up | Up | Concordant |
|  | SN | - | Down | Protein-only (PT-regulation likely) |
|  | CB | - | Up | Protein-only (PT-regulation likely) |
| Arrb1 | OB | Down | Down | Concordant |
|  | STR | - | Down | Protein-only (PT-regulation likely) |
|  | Hip | - | Down | Protein-only (PT-regulation likely) |
|  | Ctx | - | Up | Protein-only (PT-regulation likely) |
|  | Tha | Up | Down | Discordant |
|  | SN | - | Up | Protein-only (PT-regulation likely) |
|  | CB | - | Down | Protein-only (PT-regulation likely) |
| Aldh2 | OB | - | Down | Protein-only (PT-regulation likely) |
|  | STR | - | Down | Protein-only (PT-regulation likely) |
|  | Hip | - | Down | Protein-only (PT-regulation likely) |
|  | Ctx | - | Down | Protein-only (PT-regulation likely) |
|  | Tha | - | Down | Protein-only (PT-regulation likely) |
|  | SN | - | Down | Protein-only (PT-regulation likely) |
|  | CB | Up | Up | Concordant |

Up/Down indicates a statistically significant directional change; “–” indicates no significant change or not statistically significant. Concordance categories were defined as Concordant (Up/Up or Down/Down), Discordant (Up/Down or Down/Up), Protein-only (mRNA “–” with protein Up/Down), mRNA-only (mRNA Up/Down with protein “–”), and No change (“–/–”).
